# Post-discharge “continuum of care” clinical pathway (CP) for persons with severe neuro-disabilities – qualitative research to model needs-based community healthcare, capture the real-life care situation, and assess the appropriateness of the CP's concept with input from community- and hospital-based healthcare professionals

**DOI:** 10.3389/fneur.2026.1677483

**Published:** 2026-05-12

**Authors:** Stephanie Reichl, Sybille Roschka, Bernadette Einhäupl, Danae Götze, Christopher Schössow, Andreas Bender, Thomas Platz

**Affiliations:** 1 Medical Faculty, Neurorehabilitation Research Group, University of Greifswald, Greifswald, Germany; 2 Medical Faculty, Ludwig-Maximilians-Universität München, München, Germany; 3 Therapiezentrum Burgau, Burgau, Germany; 4 Department Neurorehabilitation, Medical Faculty, University Augsburg, Augsburg, Germany; 5 BDH-Klinik Greifswald, Institute for Neurorehabilitation and Evidence-based Practice, An-Institut University of Greifswald, Greifswald, Germany

**Keywords:** clinical pathway, community-based neurorehabilitation, continuum of care, healthcare, trans sectoral cooperation

## Abstract

**Objective:**

To document opinions and experiences of representatives from three different interest groups regarding “needs-based healthcare” of people with severe neuro-disability requiring home-based specialized intensive care nursing (HSICN) and continued community-based rehabilitation efforts, to evaluate the appropriateness of an evidence- and guideline-based clinical pathway (CP) for trans-sectoral specialist support by regional outreach follow-up teams (ROFT) from inpatient neurological early rehabilitation (NER) and its implementation.

**Methods:**

Qualitative exploratory study design: Semi-structured group-interviews with representatives of the three stakeholder groups, i.e., HSICN, therapists in the community (THER-C), and ROFT followed by a multi-stage analysis and interpretation process of their responses.

**Results:**

Three interviews-sessions were conducted with a total of 11 group-representatives. A total of 301 individual responses (i) were documented. Based on their experience, the interview partners identified a multitude of relevant aspects for an appropriate needs-based healthcare (i = 80) of the specific clientele, as well as facilitators (i = 44) and barriers (i = 82) for its implementation. The appropriateness of the developed CP was largely confirmed (i = 20). Support by a ROFT (i = 33) was mostly positively evaluated. Additional aspects considered necessary for needs-based healthcare (i = 42) were articulated.

**Conclusion:**

The proposed and implemented clinical pathway as a model of “needs-based healthcare” in the community for people with severe neuro-disability and the role of ROFT as an enabling specialist function supporting community healthcare were considered appropriate. The qualitative research identified several system-level constraints as barriers. Some were context-specific, i.e., remuneration for coordination time, fragmented funding streams, and incentives around decannulation/weaning. As presumably universally relevant design principles for the trans-sectoral care approach were identified: multiprofessional workforce capacity and minimum competency standards in the community, center-based specialist support for the community, case management, structured interprofessional exchange, shared documentation, and a potential role of telemedicine. The absence of patient and caregiver perspectives is a relevant limitation to be overcome by future research.

## Introduction

Because neurological disorders are the leading cause of disease burden worldwide, the number of disability-adjusted life years (DALYs) is increasing worldwide (1). For this reason, effective rehabilitation strategies are required. Post-acute inpatient rehabilitation is often sufficiently established.

However, the care situation for a continuous treatment concept in the community often deviates from the recommended scope due to numerous barriers (2). A major problem is the failure of the interface between discharge from inpatient care and the transition to community-based care. Furthermore, the various professions involved, such as therapy, nursing, and medical care, now work in a fragmented manner instead of in a transdisciplinary team. The necessary specialist assessment and diagnosis now takes place on an outpatient basis with significant delays. There are bottlenecks in medical aids and a lack of interdisciplinary care coordination (3, 4). This situation is further exacerbated for the healthcare of people with more severe and complex neurological conditions and for people living in rural areas (5).

Patients with severe neurological diseases are often mechanically ventilated or dependent on a tracheal cannula (TC). Around 35% of patients are unable to wean off mechanical ventilation during inpatient neurological early rehabilitation (NER). Even more frequently, namely in around 46% of patients who require a TC in the acute stage of the disease, this is still required on discharge due to severe dysphagia with a risk of aspiration (6). Patients with TC and/or ventilation are discharged from inpatient care when they no longer require acute inpatient treatment and short-term rehabilitation goals can no longer be achieved. As these patients cannot be cared for in regular nursing homes, home-based specialized intensive care nursing (HSICN) have been established in Germany to provide care in an outpatient setting. HSICNs care for patients either in specialized residential groups or directly in their own homes. In Germany, the number of ventilated outpatients rose from 5,000 in 2005 to 15,000 in 2018 (7).

The remaining recovery potential of these people with more severe and complex neurological conditions can only be achieved with long-term rehabilitative support. After discharge from the NER, these patients are often cared for in the community by professionals who have limited or no experience and qualifications in neurorehabilitation. To make matters worse, healthcare professionals in the community usually work on a mono-professional basis and are not networked. As a result, the need for continuous neurorehabilitation to exploit the potential for long-term recovery is frequently inadequately met while only HSICN and medical care are sufficiently provided.

“Needs-based care” in the sense of a continuum of care means that care is adapted to the needs of individual patients, primarily medical needs while including addressing the community-based organization of such care involving various multidisciplinary stakeholders. These stakeholders are then required to provide expertise in treating this clientele with a focus on TC-ventilation care, disorders of consciousness and motor functions. Furthermore, access to swallowing diagnostics, the timely provision of assistive devices, medication review, case management, and structured communication among stakeholders are necessary for needs-based care for this specific patient group (8). To ensure needs-based care and long-term improved treatment outcomes within a continuum of care, depicted in the developed CP (8, 9).

At this point, the question arises as to how needs-based care, requiring a multi-professional stakeholder team, can be implemented in the reality of community-based care, where, due to structural limitations, stakeholders often work in a fragmented and separate manner.

When looking at the definitions of team constellations, one thing is striking: although the often-described model of a multi-professional team includes the various specialist areas, each specialist area pursues its own therapeutic goals, maintaining traditional professional boundaries—this corresponds closely to the reality of community-based care. The interdisciplinary team agrees on goals with a coordinating team leader but then pursues these goals separately in terms of therapy. This variant is more in line with needs-based care but ideally requires overcoming traditional professional boundaries and mutual support in the sub-goals of the various specialist areas. This results in even more intensive and closer cooperation what is then called a transdisciplinary team (10).

The innovation project OptiNIV (Optimization of post-hospital intensive care for neurological patients) (11) addresses precisely this point. In this project, Regional Outreach Follow-up Teams (ROFTs), consisting of physicians, nurses, and therapists from three participating regional NER centers in Bavaria, Germany, follow patients for 1 year after discharge, acting as neurorehabilitation experts to provide community care providers (HSICN, therapists in the community (THER-C), physicians) with individualized, person-centered care recommendations to support functional recovery with the goal of reducing the need for HSICN. The OptiNIV innovation project stands for the establishment of a cross-sectoral healthcare organization with broad regional coverage that offers a high level of expertise to the community healthcare system while implementing an individualized, person-centered approach.

Within the project as a “continuum of care” approach, the ROFTs not only have the task of promoting medical care in terms of mechanical ventilation and TC management, but also the additional task of initiating a multi-professional approach and implementing an individual but comprehensive neurorehabilitation approach based on their expertise. To support this task, a guideline- and evidence-based clinical care pathway (CP) for the joint care of severely neurologically affected patients has been developed for use by ROFTs. The CP describes medically appropriate and necessary content for a person-centered neurorehabilitation approach with reference to multiprofessional interdisciplinary collaboration possible in the community setting, specifically for the clientele (8). This is to ensure that the treatment of a disease is standardized (12, 13). For the development of the CP and user-friendly practical implementation aids in checklist format, international evidence-based guidelines were used on the topics of neurological rehabilitation in severe disorders of consciousness (14), non-invasive and invasive ventilation as therapy for chronic respiratory insufficiency (15), prolonged ventilation weaning in neurological-neurosurgical early rehabilitation (16), positioning therapy and early mobilization for the prophylaxis or therapy of pulmonary dysfunction (17) and neurogenic dysphagia (18). Accordingly, the CP intended to reflect the desirable scope of healthcare.

As part of the project, the various healthcare providers in the community became familiar with the CP developed as part of the trans- sectoral and interdisciplinary collaboration. In semi-structured interviews, these healthcare providers and ROFT representatives were asked about their considerations for needs-based care for these severely affected neurological patients in general, any barriers or facilitators for the implementation of such aspects, the appropriateness of the CP, and the specific experiences made with the support offered by the ROFTs.

Complementary to the first round of interviews with ROFT members regarding the conceptualization of the CP and experiences made during its implementation (8), this second round of interviews now focused on the views and experiences made by healthcare professionals from the community.

The findings of the qualitative research are of interest worldwide to a wide range of stakeholders, including healthcare professionals with both a hospital and community healthcare background, the public, people with severe neurological disabilities, health insurance providers and policymakers – in short, all stakeholders involved at the interface between inpatient and community-based care and the continuum of care for the patients to be treated.

## Methods

### Study registration, ethical approval by institutional review board, and informed consent

The study protocol received ethical approval from the ethical committee by the University Medicine Greifswald (Reg. No. BB 120/24) in August 2024.

All participants (interview partners) were provided with oral and written information about the qualitative research project and gave written informed consent prior to the interviews held.

The main trial OptiNIV has been registered in the German Clinical Trials Register (DRKS) since 18 January 2022 with the ID DRKS00027326 (11).

### Clinical pathway documents

The CP used consists of a series of tools, including a patient record as a central element for shared interprofessional documentation at home. The detailed description of the components has already been extensively described (8).

### Study design

As part of the qualitative research, a theory based on focus group interviews regarding optimal, needs-based care for severely neurologically affected patients requiring HSICN was developed and compared with the implemented CP (8). In doing so, we adopted a post-positivist epistemological lens (19). The CP is intended to support the implementation of evidence-based practice recommendations [based on quantitative research (12)] in the project. This requires contextualization (20), the success of which the qualitative research approach was considered instrumental in promoting. The research perspective included modeling optimal needs-based care and comparing experiences within the project context (11) for implementation in Germany (and potentially elsewhere). In this context, the perspectives of stakeholders from the various professions in the community healthcare context, as well as the NER-based ROFTs involved in the project, were surveyed. Experiences from the actual healthcare situation in the community were used to model the theoretical (and more generally applicable) concept of optimal, needs-based healthcare and compare it with the implemented CP (8). Therefore, the research can also be considered a “grounded theory” approach (21). The subsequent coding evaluation and comparison with CP corresponds to a thematic analysis.

### Participants

#### Eligibility criteria

In the interest of targeted sampling, only health professional stakeholders from the community and representatives of multiprofessional teams who had practical experience with the project (11) and the CP (8) implemented in the community were interviewed. This is intended to harmonize optimal, needs-based care across teams and sectors (and thus across contexts), thus supporting practice-relevant modeling.

#### Exclusion criteria

Healthcare professionals from the community and representatives of multi-professional teams without own practical experience with the proposed CP (8).

#### Recruitment

Volunteer representatives of the three independent ROFTs, as well as nurses from the HSICN and THER-C), who participated in the OptiNIV project met the criteria. Each multiprofessional ROFT from NER includes physicians, nurses, and therapeutic professionals. The interviews were conducted in three small groups (three to five people with different professional backgrounds) (focus group interviews). This was intended to promote the integration of different experiences and perspectives and thus the equality and diversity of the information collected. The sample was based on the first round of interviews (8). There, the results reached saturation point with a comparably large group due to similar statements across the different professional groups. Therefore, the sample was set up to be approximately the same size with a similar ratio between representatives from nursing and therapists.

### Study procedures

After approximately 2 years of project experience and contact with the CP, three two-hour digital interviews (via video call) were conducted with the representatives of the ROFT, HSICN, and THER-C. The interviews were followed by a multi-stage qualitative analysis and interpretation process, which included 1) transcription of the interviews, 2) an initial review of the transcripts, 3) by open coding the derivation of emerging response categories and 4) the creation of corresponding “codes” (with anchor examples) in a codebook and the 5) identification of unique interview responses in the interview transcripts (and their code categories). 6) Consensus finding in cases of dual coding and 7) the summary of the content of unique responses for these categories as well as the derivation of themes were carried out separately for the three participating professional groups: ROFT, HSICN, and THER-C was following. Then 8) Cross-group thematic syntheses were derived.

### Data collection, conducting the focus group interviews

#### In advance

Basic explanations of the goal, content, and procedure as well as the desired active participation of everyone in the interview, including the opportunity to clarify questions; clarification of the interview participants, provision of written informed consent, scheduling.

#### Interviews

The interviews were conducted by two researchers (SR & TP) via video call in three group sessions, with an interview protocol and video recording. The interviews took place on three dates between 22 October and 4 November 2024. The researchers developed the CP and are qualified and have clinical experience for the type of healthcare addressed. After conducting the first round of interviews (8), the interview technique was now again used for the current follow-up round.

At the beginning, the purpose, content, process, and the desired active participation of all participants in the interview were explained again, and questions were provided for clarification. The characteristics of the interview participants (age decade, gender, occupation, and years of professional experience in neurorehabilitation) were subsequently documented. The content-related questions of the interview were then systematically queried (qualitative research) (Table 1). During each interview, each question was always answered or supplemented in the same order by the three professional groups–first HSICN, then THER-C, and finally ROFT. After the questions were answered, the interview concluded.

**Table 1:** Semi-structured interview script.

Dimension	Question
Modeling optimal needs-based care	1. Description of **optimal needs-based care** for severely neurologically affected patients requiring outpatient intensive care (ROFT) (Necessary medical and organizational content? Possible implementation from a practical perspective and experience?) (“ice breaker”)
Care in the current practical context	2. **Barriers** of the current implementation of this needs-based care for severely neurologically affected patients requiring outpatient intensive care (ROFT) 3. **Facilitators** of the current implementation of this needs-based care for severely neurologically affected patients requiring outpatient intensive care (ROFT)
***Information by the research team** (the treatment approach/CP in the OptiNIV project/individual treatment needs & goals/joint networked treatment in the team)*	
CP contents	4. Description of **needs-based care** for severely neurologically ill patients requiring home-based specialized intensive care nursing (HSICN) as outlined **in CP** (medical content and organizational aspects? What is positive and negative?) 5. Experiences made with **support from the ROFT and the CP's tools** in the OptiNIV project (Was this able to support needs-based care in terms of medical content and organizational aspects? What is positive and negative?) 6. Consideration of previously **unaddressed aspects** for needs-based care of severely neurologically impaired patients requiring outpatient intensive care (ROFT) (medical content and organizational aspects)
Prompts	- What are your thoughts on this question? - Do you have any additional content to add to what the previous speaker said? - Would you like to add any further points?

### Data analysis and outcomes

1) The interviews were transcribed verbatim. All interviews were digitally recorded and transcribed offline by RS and transcripts independently validated by SR. Transcriptions were formatted in three columns with the transcript entered in columns one and two (column one: HSICN/ ROFT/ THER-C code and question addressed, column two: line numbers and text, column three: coding of interview partner responses).
2) An initial review of the transcripts followed. The initial review served as an overview of the interview results and as preparation and orientation for the subsequent qualitative analysis steps.
3) By open coding, diverse aspects of contents of responses were deduced (emerging *post hoc*) from the interview material by the two researchers (TP & SR). These codes were meant to represent different distinguishable thematic aspects of interview content that may be relevant for modeling optimal, needs-based care, its facilitators and barriers, and the representation of actual healthcare practice. These may recur with different expressions and wording across individuals or interviews.
4) The “Codes” were summarized in a codebook. Codes were described in terms of content, given a 1–3- word name and code number and illustrated in more detail with plain text anchor example(s).
5) The specific responses from interview partners were identified. Two independent assessors (RS & SR) evaluated the transcribed interview text for unique responses that were identified and marked together with the appropriate specified code; i.e., the code name and number as well as text line [beginning of the related transcribed text] were documented in one column with marking of the corresponding text section in the separate adjacent main column of the transcript. The line number of the text can be taken as an independent variable, and the unique response and related code as a dependent variable.
6) When ratings by the two independent assessors were not in agreement, the rating was discussed in group meetings together with a third rater (TP), and final coding agreed on.
7) For each specified content aspect (i.e., code), the content of the unique responses given by interview partners were then summarized. After that the summarized content (per code) was collated within “themes”. The results were analyzed by comparing the three interviewee groups: HSICN, ROFT, and THER-C. Subsequently, a model for an optimal needs-based healthcare and framework recommendations for its implementation were derived, considering the facilitators and barriers.
8) From the themes derived, cross-group thematic syntheses were performed. This includes the derivation and verification of the summaries in the appendices using the raw data.

### Data management

The digital video files of the interviews were stored on two project-specific Veracrypt-encrypted hard drives (original & copy) in restricted access workrooms of the neurorehabilitation research group (NRG) of the University Medicine Greifswald for the duration of the OptiNIV project (until 07.2025) and was deleted afterwards. The transcribed text contains only anonymous information and will be stored for a longer period, also for the purpose of later secondary analyses by the NRG. Furthermore, the ratings and markings of the raters were entered into the text files (1 file per rater and a file for agreed (final) codings).

### Statistical analyses

As the research was planned as qualitative research, no hypothesis-testing statistical analysis was intended. While exploring thematic content aspects emerging from interview responses was the focus of analysis, descriptive statistics on type and frequency of codes and as clustered into emerging themes were provided as some additional aid for interpretation. *A priori* sample size collection was not indicated (no hypothesis testing).

## Results

## Development of coding and categorization

The three interviews were conducted with a total of 11 individuals, consisting of representatives from the three ROFTs (1 physician, 1 speech and language therapist, 1 occupational therapist) as well as representatives of the HSICN (1 psychologist; 4 nurses) and THER-C (3 speech and language therapists) reflecting diversity of age, profession, and experience in neurorehabilitation. Details about the participant description are given in Table 2.

**Table 2:** Description of study participants (*n* = 11).

Participant	S	AD	Profession	BG	PYN
1	m	4	Nurse	HSICN	28
2	f	3	Speech- and language- & respiratory therapist	THER-C	8
3	m	4	Occupational- & respiratory therapist	ROFT	13
4	m	4	Nurse (specialist for intensive care medicine) & Nursing service manager	HSICN	22
5	w	4	Psychologist and Managing Director	HSICN	10
6	m	1	Nurse & respiratory therapist	HSICN	8
7	f	4	Speech- and language therapist	THER-C	28
8	f	4	Physician	ROFT	11
9	f	4	Nurse & residential area management	HSICN	9
10	f	2	Speech- and language therapist	THER-C	11
11	f	1	Speech- and language therapist	ROFT	9

S (Sex): f = female, m = male; AD (age decade): 1 = 18–29 years, 2 = 30–39 years, 3 = 40–49 years, 4 = 50–65 years; BG = background; PYN = Professional years neurorehabilitation; HSICN = home-based specialized intensive care nursing; THER-C = therapists from the community sector; ROFT = regional outreach follow-up team.

During the first step of qualitative data analysis of the interviews, 38 distinguishable emerging content aspects (codes) were identified and operationalized in a code manual and grouped into five thematic fields with a total of 10 thematic code categories to be seen in Supplementary Table 1.

The five thematic fields first include the description of the various stakeholder groups for appropriate needs-based healthcare (1) for the described patient group in the community setting. Based on this, aspects for the implementation (2) of the described needs-based healthcare were presented. In the third thematic field, the appropriateness (3) of the developed clinical pathway as a concept for the support of needs-based healthcare was reflected. Subsequently, aspects of ROFT's support (4) for the community stakeholders in the implementation of needs-based healthcare were shown. In the fifth thematic field, additional aspects (5) of needs-based healthcare not covered within the other thematic fields were supplemented.

The ten thematic code categories include, for example, observed “facilitators” and “barriers” in the community healthcare setting, but also positively or negatively perceived aspects with regard to the appropriateness of the CP's conceptualization or the ROFT support (medical and organizational aspects).

## Thematic processing of the transcript statements

The interview transcripts were then analyzed in detail by two independent reviewers (SR & RP). A total of 301 specific unique responses from the interviewees were identified.

Example statements from the English-language version of the interviewees are presented below. The original German transcript for example statements assigned to the key statements for all thematic code categories can be found in Supplementary Tables 2–4, sorted for the three groups: HSICN, THER-C, and ROFT.

From these, 187 summary statements were derived. An overview of these summary statements can be found in Supplementary Table 5 for each thematic aspect synoptically presented for the three interview groups: HSICN, THER-C, and ROFT.

These responses were marked in the transcripts along with the interviewee's profession and assignment to one of the stakeholder groups (HSICN, THER-C, or ROFT), and the corresponding code in the manual. In accordance with the principle of equal treatment and diversity in data collection, all responses were considered informative. From the 187 summary statements of the three groups HSICN, THER-C and ROFT 29 cross-professional core statement collections were deduced (Table 3).

**Table 3:** Overview–Summary statements from the three stakeholder groups (HSICN, THER-C, ROFT) on the 5 thematic fields and 10 categories.

Thematic field	Description of an appropriate needs-based healthcare	
*Thematic code category*	*Content-related aspects*	•Nurses: Adequate training of nursing staff (including continuing education) and specialized intensive care nurses. •Therapists: therapeutic care including occupational therapy, speech therapy, physical therapy, psychology, respiratory therapy, and nutritional counseling. •Physicians: Provision of GP and specialists (neurologists, urologists, ENT specialists, anesthesiologists, dentists). •Technical aids Appropriate TC management, FEES, monitoring, and flexible interventions as needed. •Medications: Medications are tailored to the patient's current needs.
*Thematic code category*	*Organizational aspects*	•Nurses: 1-to-3-patient ratio by qualified nursing staff; centralized care organization; intensive care independent of TC/ventilation. •Therapists: various professions with sufficient treatment frequency; interprofessional scheduling of therapies with appointment schedules; provision of regular treatment reports; remunerated interprofessional exchange of treatment goals. •Physicians: provision of additional training in outpatient intensive care support, timely provision of specialist care with diagnostic interventions, where possible through home visits. •Technical aids: use of telemedicine as a care resource, assistive device organization by the pre-treatment (rehabilitation) hospital. •Networking: good interdisciplinary teamwork between nursing staff, physicians, therapists; case managers, assistive device providers, and specialized outpatient follow-up teams; good team leadership in shared living communities; involvement of family members; better coordination and delegation of decision-making authority for outpatient healthcare staff; regular professional development for outpatient professionals; regular team meetings; establishment of permanent treatment teams; local hospitals with qualified care for the most critically ill. •Financing: Coverage of costs for interdisciplinary communication, intensive patient preparation, and team training; financing options for the continuation of intensive care after successful weaning/decannulation.
Thematic field	Implementation of needs-based healthcare	
*Thematic code category*	*Facilitating aspects*	•Nurses: high quality of care; identification of individual patient resources; hiring additional nursing assistants. •Therapists: high-quality therapy from all disciplines for this patient group; guided therapy exercises by nurses to increase therapy time. •Physicians: specialist medical care through telemedicine; issuance of treatment prescriptions by all disciplines possible. •Networking: interdisciplinary collaboration between all professions involved in patient care in the outpatient sector; measures to support teamwork and communication; team leadership in shared living arrangements with leadership and management skills; delegation of tasks to specialists. •Financing: cost-effectiveness through centralized organization and patient accommodation in the HSICN for nursing and therapists.
*Thematic code category*	*Barriers*	•Nurses: No central contact person in the HSICN; difficulties recruiting new staff; inadequate qualifications of nursing staff. •Therapists: Lack of therapists; lack of therapist qualifications in ventilation/TC; insufficient treatment frequency; inadequately compensated for the time spent on documentation. •Physicians: Insufficient qualifications in ventilation/TC, lack of specialists; no home visits for patients in HSICN; no swallowing diagnostics in the community. •Technical aids: Necessary care is hardly possible due to restrictive health insurance approval procedures. •Medication: Inadequate medication prescription and adjustment. •Networking: Hospital admissions to local, underqualified hospitals are risky; weaning attempts are hardly feasible without an interdisciplinary care team in the community. Insufficient treatment delegation for outpatient specialists by physicians. Lack of coordination of interprofessional collaboration. Inefficient overlap between therapy sessions and nursing activities in everyday life. Different documentation systems exist across professions. •Financing: Cost negotiations with payers are ineffective; necessary unpaid additional services mean that HSICNs do not cover their costs; patients are not decannulated for financial reasons despite medical possibilities due to financial mis incentives; HSICNs incur financial losses when referring patients to hospitals due to the requirement to keep beds free; the quantity of necessary care supplies is not sufficiently co-financed; Travel allowances for therapists' home visits are too low; home visits by physicians in HSICNs are not adequately refinanced; the bureaucratic documentation and billing burden is too high
*Thematic code category*	*Implementation reflection, clinical pathway/Positive aspects*	Description of patient-centered collaboration between different professional groups; uniform documentation system for all professional groups; easier access to patient information for all professional groups; team meetings could take place online via video conferencing.
*Thematic code category*	*Implementation reflection, clinical pathway/Negative aspects*	Too much handwritten and duplicate documentation; the clinical treatment pathway and patient records are inadequately implemented in daily practice.
Thematic field	ROFT support for needs-based healthcare	
*Thematic code category*	*Positive aspects*	Support with valuable advice and opportunities for interprofessional exchange; diagnostics and follow-up examinations (i.e. FEES) can be performed directly on an outpatient basis at HSICN; good telephone accessibility for inquiries; provided resources and materials (such as the patient file) were helpful; possibility of inpatient admission as part of the Interdisciplinary structured inpatient assessment (ISSA) and Neurological Interval Rehabilitation (NIR); ROFT has sufficient time for project visits
*Thematic code category*	*Negative aspects*	ROFT inefficient despite good HSICN care; replacing daily, time-consuming project calls with emails; inadequate use of provided resources and materials (such as patient folders) due to difficult access; suboptimal coordination and organization with ROFT; ROFTs were assigned to many scattered HSICNs; first ROFT visit conducted earlier than after 4 weeks.
Thematic field	Additional aspects for needs-based healthcare	
*Thematic code category*	*Medical aspects*	Utilization of every potential for improvement for patients; measurement of treatment success must be qualitative, independent of weaning/decannulation; provision of patients with additional therapies such as osteopathy is sensible; mandatory standards for the safety of ventilated and cannulated patients.
*Thematic code category*	*Organizational aspects*	Patients prefer care in HSCIN rather than in the hospital; also conduct interviews with patients about care; HSICN desires further study projects for severely affected patients; HSICN would like to help shape patient care in the case of hospital admission in exchange for funding; continuing education programs and supervision for nursing staff could counteract the shortage of skilled workers; implementation of a cross-sector and cross-professional case manager for patients with severe neurological disorders is advisable; implementation of outpatient therapist networks for improved patient care in HSICN is advisable; continuation of the OptiNIV study for patients in HSICN is desired.

HSICN = home-based specialized intensive care nursing; THER-C = therapists from the community sector; ROFT = regional outpatient follow-up team; GP = general practitioner; TC = tracheal cannula.

During the interviews, a strong convergence was found between the stakeholder groups HSICN/THER-C/ROFT on the core requirements for needs-based care and their representation in the CP.

After extensive code generation of various aspects and coding, it was then possible to recognize that very similar aspects relating to needs-based healthcare, facilitators and barriers to implementation and the concept of CP were mentioned by all three stakeholder groups independently of each other. It can therefore be assumed that the content of the results was saturated by the representatives of the three groups.

The following aspects were entertained by the interview partners:

Important content-related aspects included the existence of an inter- or transdisciplinary team with all the necessary professions in nursing, therapy (physiotherapy, occupational therapy, speech therapy, psychology, respiratory therapy, nutritional counseling), and physicians (general practitioners and relevant specialists). All team leaders were considered to require appropriate specialization and further training for the patient clientele under investigation. Furthermore, technical equipment for swallowing diagnostics and monitoring was considered essential.

In addition, organizational aspects such as a nursing care ratio of 1:3 by qualified nursing staff with centralized nursing organization were reported as important. The provision of intensive nursing care should be possible regardless of ventilation/TC due to the severity of the underlying neurological condition. The various therapeutic professional groups should be available with sufficient treatment frequency. Interprofessional therapy planning with remunerated time for interprofessional exchange of treatment goals and interventions was considered necessary. All physicians involved should receive further training in community-based care of the served clientele, and prompt specialist care with diagnostic interventions, if possible, through home visits, was reported to be important. Telemedicine should be used as a care resource, and the organization of aids should be centrally controlled and monitored by specialists (from NER centers). Well-functioning inter- or transdisciplinary cooperation should be developed within the large stakeholder network including nurses, physicians, therapists, case managers, medical aid providers, relatives, and specialized outpatient aftercare teams.

The interview partners articulated main barriers for implementing the needs-based care. Obstacles for inter- or transdisciplinary teamwork and networking were identified. Referring HSICN patients to local, underqualified hospitals was considered risky. Withdrawal attempts (TC/ventilation) were considered hardly feasible without an interdisciplinary treatment team in the community. Regulations for the delegation of procedures to specialized therapists in the community by physicians were characterized as insufficient. The absence of central coordination and communication of interprofessional collaboration was mentioned as a major obstacle. Inefficient overlaps between therapy sessions and nursing activities in everyday life might led to suboptimal patient care. The different documentation systems used by the various professional groups were experienced as not compatible, resulting in duplicate documentation.

In the area of nursing, a lack of key contact persons in the HSICN was observed. Difficulties in recruiting staff and nursing staff who was considered inadequately qualified were entertained. In the area of therapy, a substantial shortage of therapists, with an additional lack of qualifications in ventilation/TC was reported. The available treatment frequency was evaluated as insufficient, and the remuneration for documentation work reported to not cover costs. In the area of medical care, a lack of qualifications in ventilation/TC, and a shortage of specialists willing to make house calls to patients in the HSICN were mentioned as barriers. It was complained that no swallowing diagnostics were available in the community.

## Discussion

### Clinical relevance of the research topic

With the advances of intensive care more critically ill persons survive, but suffer from severe neurological deficits caused by either a Post-Intensive Care Syndrome (PICS) or a primary neuro-condition (such as stroke or traumatic brain injury) (22). Despite the high success rate of inpatient neurological early rehabilitation (NER), a certain proportion of severely neurologically ill patients continue to require intensive nursing care after discharge from NER due to continued TC/ventilation requirements and constant qualified medical monitoring (6). In this situation, home-based specialized intensive care nurses (HSICN) and the responsible general practitioners provide the necessary basic medical care in the community. However, specific neurorehabilitation needs are not met, as this expertise is available only in regional NER centers, not in the community. Within the OptiNIV project, the HSICN and therapists in the community (THER-C) involved in patient care received specialist support by multidisciplinary outreach teams (ROFT) located in one of the three participating regional NER centers (11). This study presents the views of the three stakeholder groups HSICN, THER-C, and ROFT on the healthcare situation of these patients, their care needs, experiences made with the trans-sectoral healthcare approach, and demonstrates consensus of views of community- and hospital-based stakeholders on various themes to such care.

### Model of needs-based healthcare

The interview partners identified three transferable principles for the cross-sectoral and inter- or transdisciplinary needs-based and person-centered healthcare as a continuum of care:

Firstly, this includes the need for an inter- or transdisciplinary rehabilitation team in the community (itself), which is made up of a local network of all relevant healthcare providers for an individual patient's healthcare needs and can adapt evidence-based treatment decisions to the local situation and circumstances of an individual patient.

Secondly, the various community healthcare professionals' benefit from specialists from neurorehabilitation centers who offer clinical support, supervision, and training (2, 23, 24). Telemedicine support from these specialists is also considered a relevant option (25–28). The specialist teams from the inpatient teams should by no means replace the therapeutic care capacities in the community but rather act as a professional supplement for specific healthcare needs of this patient clientele.

Thirdly, the trans-sectoral cooperation between the local network in the community and experts from the inpatient facilities needs to be structured. It would be advantageous to have an individualized case management plans, ideally with a case manager in charge who can structure interdisciplinary communication. A joint cross-sector and cross-disciplinary documentation system promotes trans-sectoral teamwork (8, 29–32). To enable the collaboration as a team, the necessary framework conditions need to be created at system-level (9). There are already several promising research approaches that could be utilized and transferred to standard care (33–38). In joint teamwork involving hospital-based and community-based healthcare professionals, rehabilitative goals are identified together with persons with disabilities and their carers and worked on together in accordance with the biopsychosocial model (39). Such a healthcare approach could be highly effective by adapting treatments to patients' needs, utilizing competencies available in such extended trans-sectoral teams while at the same time ensuring that team members have the knowledge, skills, and experience required for their respective areas of responsibility and recognizing and managing the uncertainty and complexity of individual patient care (40, 41).

### System-level limitations on the implementation of needs-based healthcare

As part of the implementation study, numerous systemic limitations were identified by the participating stakeholders.

In Germany, public reimbursement schemas can act as incentive against decannulation/weaning. While these patients remain severely neurologically impaired after a successful decannulation/weaning, their care allocation changes from an HSICN setting (receiving more funds) to a regular nursing home (receiving much less funds) with the consequence of less individual resources for their care being available.

Other identified system-level limitations are not related to the specific German context and indicate barriers that would have to be addressed in many different regional contexts.

A significant and growing shortage of qualified staff and personnel with insufficient specific qualifications to serve people with severe neuro-disabilities is considered a major barrier. Case management and interdisciplinary exchange and its shared documentation are not funded or remunerated and therefore rarely take place.

Patients receive little specialist care and diagnostics, as specialists are rarely able to make house calls to specialized care facilities and patients are not mobile enough to attend outpatient appointments. At times, necessary medications were not sufficiently available on prescription and were not adequately adapted to the changing needs of patients. Hospital stays were sometimes considered more risky than beneficial for patients, as nearby basic level hospitals may not have been adequately prepared for the special needs of this patient group.

The barriers to the implementation of the CP and needs-based healthcare described above thus point to system-specific limitations with international relevance.

If health policy does not create a basis on which the three cornerstones of needs-based care of this clientele—i.e., an inter- or transdisciplinary rehabilitation team in the community, the involvement of specialists from neurorehabilitation centers in community healthcare, and trans-sector teamwork/cooperation between the local community network and experts from inpatient facilities — demand-oriented, person-centered care is hardly feasible for this specific patient clientele.

Further, healthcare capacity building is key, as such specialized care highly depends on the available resources in terms of skilled personnel (26–28). Hospitals might be able to support specialist input to community networks but they cannot substitute for a lack of community-based rehabilitation teams to start with.

### Reflection on research methods used and imitations of the results

The researchers themselves were independent data analysts for the main OptiNIV project (11) from a different federal state than the actual study location and thus acted as independent researchers with comparable clinical backgrounds. The authors were therefore not part of the intervention team and thus had no conflict of interest regarding the interview content or its coding. Various protective measures were implemented during the analysis. The qualitative analysis of the responses and the independent coding were conducted openly and identified new content and themes based on the experiences, insights, and reflections provided by the respondents. Methodologically, both a rigorous multi-step qualitative research process based on consensus of two or more researchers and the consistency of responses across teams add to the credibility and transferability of findings to other similar healthcare situations. In the event of disagreements regarding code assignments, a consensus was reached in joint discussions. Frequency counts (for codes) were given as descriptive aids only (not for weighting perspectives and their relevance).

Only three ROFTs took part in the OptiNIV project (11) and thus only representatives of these teams could therefore be interviewed. More than 57 outpatient care services were integrated in the project; the exact number of participating community-based therapists could not be estimated. The 11 interview partners consisted of volunteers from these three sectors. However, since the interview participants, as representatives of their professions, independently and across interviews expressed very similar aspects regarding the modeling of needs-based care, facilitators and barriers to implementation and experiences with the CP, it can be assumed that the results are saturated.

The qualitative interviews were conducted with representatives of the three teams from three neurorehabilitation centers and the community-based professionals that provided cross-sectoral care in Bavaria, Germany's largest federal state. Although the interviews are representative of the region covered (i.e., not bias by one specific context), regional bias cannot be ruled out.

Furthermore, this follow-up second interview study was unable to include the perspectives of patients and relatives. This would be desirable content for future research work within the framework of, for example co-design, interviews with relatives and patients, or validation using mixed-methods design with an implementation evaluation.

In addition, the severely affected patient group treated in this project is less common than major neurological treatment indications in the community such as hemiparesis after stroke; the healthcare situation and pre-requisites for an inter-sectoral network for more frequent conditions might well be different.

Finally, as this work is qualitative research, no claim is made as to the representativeness of the data and no hypothesis testing was intended.

### Health policy and practical consequences

This qualitative research demonstrates to health policymakers that a hybrid approach combining collaborative center-based and community-based healthcare for patients with highly specialized healthcare needs can be a model for addressing specific needs with the potential to promote community healthcare. Although subjective, the description of needs-based care as well as the reported experiences with practical implementation of the proposed CP in everyday life, underpin the care reality for this patient group including barriers and facilitators for appropriate healthcare. The use of such insights by health policymakers has the potential to reduce barriers for appropriate healthcare, and thus promote the implementation of needs-based healthcare in practice and reduce the burden of inpatient consultations and treatments.

This research paper primarily focuses on the perspective of community-based stakeholders in patient care. In combination with the additional views of inpatient providers in both this research and in the preceding paper (8), it provides a comprehensive presentation of the supportive as well as the numerous inhibiting system-level conditions based on which health policy changes should be made to ensure a sufficient quality of patient care.

The absence of patient and caregiver perspectives is nevertheless a relevant limit of this research to be overcome by future research.

## Conclusions

The qualitative research contributed to our knowledge about the design of needs-based care of people with severe neuro-disabilities requiring intensive nursing care in the community, as well as the current state of practical implementation and the relevance of a proposed CP in this context. The CP is grounded in guideline-oriented thinking and translated into operational elements.

The interviewed stakeholders largely endorsed the CP conceptually while identifying actionable implementation constraints.

An inter- or transdisciplinary rehabilitation team in the community is (not existing while) needed, which should be made up of a local network of all relevant healthcare providers for an individual patient's healthcare needs.

Community healthcare professionals benefit from specialists from neurorehabilitation centers who offer clinical support, training and the possibility of supervision.

The use of hybrid care models that make specialist expertise from hospitals accessible to community healthcare workers and their patients offer great potential for the optimal use of existing resources to support a continuum of care of patient clientele with a need for highly specialized healthcare that cannot comprehensively be met by the community healthcare system alone.

Barriers for implementation identified can be addressed by targeted solutions such as establishing frameworks, organization and funding for community-based teams and trans-sectoral cooperation, comprehensive training courses for community-based healthcare professionals supporting the acquisition of key qualifications, tele-medicine applications (for interprofessional exchange and therapy) or digital medical records to be shared and used along the continuum of care from the hospital to the community.

Future research should further examine and integrate the perspectives of both people affected by severe neuro-disabilities and their carers for a co-design of an appropriate and acceptable healthcare.

## Clinical message

- Both community-based and hospital-based healthcare professionals prioritize a comprehensive trans-sectoral and transdisciplinary medical network with central organization in terms of case management for needs-based healthcare for people with severe neurological diseases.
- This competence network can only be created through multiprofessional inter- or transdisciplinary teamwork, which requires organization, technology (incl. documentation) and funds for professional exchange and case management.
- The proposed clinical pathway (CP) adequately covers relevant clinical aspects and healthcare organization for this patient clientele.
- A practical documentation system is considered instrumental for its implementation.

## Data Availability

The original contributions presented in the study are included in the article/Supplementary material, further inquiries can be directed to the corresponding author.
