# Supplementary material for: Post-discharge “continuum of care” clinical pathway (CP) for persons with severe neuro-disabilities – qualitative research to model needs-based community healthcare, capture the real-life care situation, and assess the appropriateness of the CP's concept with input from community- and hospital-based healthcare professionals
Source: Front Neurol. 2026 May 12;17:1677483. doi: 10.3389/fneur.2026.1677483 (PMC13248884; doi:10.3389/fneur.2026.1677483)
Supplement: Supplementary file 1 [file Data_Sheet_1.pdf]

Supplementary Table 1. Overview of the 38 categories, grouped into five thematic fields, 10 code categories and 23 subcategories.

		H	T	R	Σ
**Thematic field**	**Description of an appropriate needs-based healthcare**	**38**	**22**	**20**	**80**
Thematic code category	*Content-related aspects*	*12*	*6*	*3*	*21*
Code subcategories	Nurses				
	Therapists				
	Physicians				
	Technical aids				
	Medication				
Thematic code category	*Organizational aspects*	*26*	*16*	*17*	*59*
Code subcategories	Nurses				
	Therapists				
	Physicians				
	Technical aids				
	Networking				
	Financing				
**Thematic field**	**Implementation of needs-based healthcare**	**63**	**33**	**30**	**126**
Thematic code category	*Facilitating aspects*	*33*	*7*	*4*	*44*
Code subcategories	Nurses				
	Therapists				
	Physicians				
	Networking				
	Financing				
Thematic code category	*Barriers*	*30*	*26*	*26*	*82*
Code subcategories	Nurses				
	Therapists				
	Physicians				
	Technical aids				
	Medication				
	Networking				
	Financing				
**Thematic field**	**Appropriateness of the clinical pathway for the support of needs-based healthcare (medical and organizational aspects)**	**10**	**5**	**5**	**20**
Thematic code category	*Clinical pathway conceptualization/ positive aspects*	*6*	*3*	*1*	*10*
Thematic code category	*Clinical pathway conceptualization/ negative aspects*	*4*	*2*	*4*	*10*
**Thematic field**	**ROFT support for needs-based healthcare**	**14**	**8**	**11**	**33**
Thematic code category	*ROFT support/ positive aspects*	*9*	*6*	*3*	*18*
Thematic code category	*ROFT support/ negative aspects*	*5*	*2*	*8*	*15*

Thematic field	Additional aspects for needs-based healthcare	27	5	10	42
Thematic code category	*Medical aspects*	*14*	*4*	*2*	*20*
Thematic code category	*Organizational aspects*	*13*	*1*	*8*	*22*

Explanations: Numbers: Number of entries as descriptive aids (not weightings), i.e. specific, unique responses from interview partners within the respective code (sub)categories in total and divided into the professional groups H(HSICN)-home-based specialized intensive care nursing; T(THER-C)-therapists from the community sector; R(ROFT)-regional outpatient follow-up team
