# Supplementary material for: Post-discharge “continuum of care” clinical pathway (CP) for persons with severe neuro-disabilities – qualitative research to model needs-based community healthcare, capture the real-life care situation, and assess the appropriateness of the CP's concept with input from community- and hospital-based healthcare professionals
Source: Front Neurol. 2026 May 12;17:1677483. doi: 10.3389/fneur.2026.1677483 (PMC13248884; doi:10.3389/fneur.2026.1677483)
Supplement: Supplementary file 2 [file Data_Sheet_2.pdf]

Supplementary Table 2 (HSICN). Stakeholder- Group: Home-based specialized intensive care nursing (HSICN).

Individual statements and summary statements for the five thematic fields and ten code categories.

Sequence of presentation (thematic fields):

Description of an appropriate needs-based healthcare

Implementation of needs-based healthcare

Appropriateness of the clinical pathway for the support of needs-based healthcare (medical and organizational aspects)

ROFT support for needs-based healthcare

Additional aspects for needs-based healthcare

Thematic field: Description of an appropriate needs-based healthcare Thematic code category: Content-related aspects

Examples of individual statements	Summary statement
Nurses	
• „Dann komme ich auf pflegerische bedarfsgerechte Versorgung. Da sehe ich, dass es auch Fachpersonal benötigt wird wie Atmungstherapeuten oder Fach-Intensiv-Anästhesie-Krankenpfleger, auch die Fachpflegekräfte mit Basis-, aber auch Aufbaukursen in der Versorgung.“ (N)	Needs-based nursing care primarily comprises professional nursing staff with basic and advanced training as well as specialist intensive care nurses.
Therapists	
• „Und die Menschen erwerben noch verschiedene andere Schäden im Rahmen ihrer Erkrankung, die dann alle in der ambulanten Intensivpflege auftauchen und Thema werden. Sodass hier ein interdisziplinäres Team hilft (…) Es braucht Ergos, Logos und Physios (…)“ (M)<br>• „Die Thematik Psychosomatik muss berücksichtigt werden, aus meiner Sicht, ist logisch, ist meine Baustelle. Wir übernehmen die Menschen mit ganz viel Lebenserfahrung, den verschiedenen Schäden, die wir alle einpacken im Laufe des Lebens, die hören ja nicht auf, wenn wir ein Tracheostoma haben oder beatmet sind.“ (M)<br>• „Mit dabei mit Sicherheit gehört (…) bei Menschen mit Beatmung logischerweise auch noch Atemtherapie, (…) Atemtherapeuten.“ (N)<br>• „(…) dass der Mensch danach in der Wohngemeinschaft nicht nur Klinikalltag erlebt, sondern auch wirklich wohnen kann. Dazu gehört Mobilisation, dazu gehört raus.“ (N)	A needs-based, multi-professional therapeutic care includes occupational therapy, speech therapy, physiotherapy, psychology, and respiratory therapy, which enable the patient to participate in everyday life and to take part in life.
Physicians	
• „Bedarfsgerechte Versorgung ist natürlich primär erst mal die hausärztliche Versorgung. Wie bei jedem normalen Patienten. (…) Neurologische Versorgung durch Neurologen, oder ja (...) auf jeden Fall. Mit dabei mit Sicherheit gehört bei den Patienten dann auch urologische Versorgung, HNO, weil Außerklinische und Neurologische betrifft ja meistens auch die Versorgung mit Trachealkanülen, dementsprechend HNO-Versorgung (…).“ (N)<br>• „Natürlich braucht es in erster Linie hausärztliche Versorgung, aber auch allgemein fachärztliche - nicht nur einen Facharzt für Neurologie, sondern auch (…) HNO, dann - was ganz oft vergessen wird - auch ein Zahnarzt. Wie oft kommt Zahnarzt in die außerklinische Intensivpflege? Deswegen würde ich das Verfassen als fachärztliche Versorgung zusätzlich.“ (N)	Needs-based medical care primarily comprises GP care, but also care by specialists (neurologists, urologists, ENT specialists, anaesthetists, dentists).
Technical aids	
• „Und da ist halt sowas wichtig, dass die Kanüle richtig sitzt oder dass nicht den ganzen Tag alles voller Speichel ist, weil einfach der so viel produziert, dass man das mit dem Absaugen gar nicht wegbringt. Da saugt man, und dann läuft das und läuft und läuft.“ (N)	A needs-based patient care comprises an adequate TC management.

Explanations: M - managing; N - nurse; GP - general practitioner; TC – tracheal cannula.

<u>Thematic field</u>: Description of an appropriate needs-based healthcare <u>Thematic code category:</u> Organizational aspects

Nurses	
• „(…) schlechter sollte er nicht werden. Also ein Pflegeschlüssel, der schlechter ist als 1 zu 3 geht gar nicht mit diesen Patienten.“ (M)<br>• „Nur kurz noch, organisatorisch, organisatorisch für mich /. Ja. Ich selber bin leitender Atmungstherapeut, da muss man sich natürlich auch Gedanken machen über Management, wie viele Ressourcen kann ich überhaupt einsetzen? Das und wie viele Leitungskräfte brauche ich auch, dass ich auch in der Pflege erlebe, dass manchmal auch zu viele Leitungskräfte vorhanden sind. Da würde ich Ressourcen eher sparen und dann eine andere Struktur mehr überdenken, dass es wieder in diese zentralisierte Rolle geht, mit Pflegedienstleitung, die dann oder Bereichsleitung, wo da drunter mehrere Leitungskräfte dann sind und dieses ganze Organisatorische dann übernehmen.“ (N)<br>• „Also die Versorgung in den Kliniken im Umfeld ist zum Teil hochschwierig, weil die Kliniken mit unseren Patienten einfach massiv überfordert sind.“ (M)<br>• „(…) oder uns ranlassen. Ja, also wenn wir unsere Patienten in die Kliniken begleiten könnten und dafür auch ein bisschen Geld kriegen könnten. (…) Aber es wäre eigentlich nötig, um unsere Patienten durch die zum Teil langen Klinikaufenthalte durchzuboxen, dass die heile wieder zurückkommen.“ (M)	A nursing ratio of 1(nurse):3(patients) is considered necessary to provide good nursing care for the severely affected patients.<br><br>The leadership hierarchy in nursing should be reduced to a single central role to save human resources.<br><br>Local hospitals qualified to care for these critically ill patients are needed. Alternatively, care during hospital stays could be provided in non-specialized facilities by nursing staff in shared living communities (for a fee).
Therapists	
• „Das, was OptiNIV gezeigt hat - wie kann man sich bei den Atmungstherapeuten melden oder bei Fachpersonal, wo ich Unterstützung brauche als Pflegekraft, in der außerklinischen Intensivpflege. (…) Und dass, dass die Firmen auch solche Stellen auch zentralisieren können.“ (N)<br>• „Den Atemtherapeut braucht man nicht in jeder WG, aber es hilft, wenn man ihn einfliegen kann wie bei OptiNIV (…)“ (M)<br>• „(…) es muss nicht unbedingt Atmungstherapeut sein, sondern einfach eine Fachkraft oder jemand vom Fachpersonal, der sich mit allgemein Intensivmedizin, vor allem außerklinische Intensivpflege auskennt.“ (N)	Central provision and placement of specialists such as respiratory therapists by companies can support needs-based care and should be carried out by trained specialists as needed.
Physicians	
• „(…) wie kann man diese ärztliche Versorgung in der außerklinischen Intensivpflege verbessern, wenn man Strukturen schafft, dass ein Hausarzt eine Zusatzweiterbildung für die außerklinische Intensivpflege machen kann.“ (N)<br>• „Oder ja, dass die Hausärzte da oder die Ärzte vielleicht irgendeinen Anreiz bekämen, damit sie diese Patienten eher betreuen. Weil ich habe halt so das Gefühl, dass das ja, mehr abgelehnt wird, einfach, weil es schwierig ist mit solchen Patienten.“ (N)<br>• „Ja, bedarfsgerechte Versorgung wäre wirklich kurzfristige, maximal spätestens nach einer Woche, je nach Dringlichkeit am gleichen Tag, Reaktionsmöglichkeiten.“ (N)	Physicians (especially GPs) should be given the opportunity to complete additional training in outpatient intensive care medicine.<br><br>The timely provision of specialist medical care is also part of a needs-based care.
Technical aids	
• „Vieles könnte man tatsächlich aus Distanz auch lösen. Telemedizin - das wäre eine Umsetzung.“ (N)	Telemedicine would be one method of realising needs-based care.
Networking	
• „Und auch die Zusammenarbeit mit Ergotherapie, mit Krankengymnastik, die ist auch sehr, sehr gut. Logopädie natürlich. Auch mit Ärzte. Die Neurologen kommen ins Haus. Hausarzt ist bei Bedarf täglich da.(…), das ist auch eine Voraussetzung für eine gute Pflege und Versorgung von kranken Menschen.“ (N)<br>• „Organisatorisch (…) ist es (…) wichtig, dass so viel wie möglich aus einer Hand kommt oder dass eine Hand Vieles steuert und organisieren kann. Also eine zentrale Ansprechperson, die sich dann um die weiteren Inhalte kümmert oder die weiteren Ansprechpartner vermitteln kann. (…) wenn das optimal über einen Koordinator organisiert werden kann, der als Ansprechpartner fungiert und entweder die einzelnen Fachärzte schon zur Hand hat oder dann weitervermittelt.“ (N)<br>• „(…) eine Führungsstruktur, die auch dafür sorgt, dass die Menschen interdisziplinär denken (…) Dazu braucht es wirksame Führungskräfte, es braucht wirksame Management- Systeme, die den Menschen auch klarmachen, dass die Mitarbeitenden was sagen können, wenn sie eine Idee haben.“ (M)	Good co-operation and support between nursing staff and physicians and therapists is regarded as a prerequisite for needs-based care.<br><br>A coordinator and contact person for the team in the residential community, who controls and organizes further medical care, is considered important.

• „Und es braucht ein Team, das gern unter einer pflegerischen Leitung ein pflegerisches Konzept entwickelt, das auf den Bewohner angewandt wird, wo alle zusammen an einem Strang ziehen. (…) das interdisziplinär denken kann.“ (M)<br>• „Das, was OptiNIV gezeigt hat - wie kann man sich bei den Atmungstherapeuten melden oder bei Fachpersonal, wo ich Unterstützung brauche als Pflegekraft, in der außerklinischen Intensivpflege. Und dann bekomme ich diese Unterstützung dementsprechend oder frühzeitig, sodass ich auch eine Klinikeinweisung vermeiden kann.“ (N)	Leadership and its structures in the residential community should encourage interdisciplinary thinking, the contribution of ideas and a concept-based cooperation within the nursing team.<br><br>The continuation of outpatient care by outpatient specialist aftercare teams would form a valuable addition and can help to prevent hospital admissions.

Explanations: M - managing; N - nurse; GP - general practitioner.

<u>Thematic field</u>: Implementation of needs-based healthcare <u>Thematic code category:</u> Facilitating aspects

Examples of individual statements	Summary statement
**Nurses**	
• „Man sieht einfach, wie die Patienten, die sehr schwerst Kranke, sind dankbar und ein Lächeln von denen oder ein Danke (unv.) die einfach nicht sprechen oder reden können und sagen dich Dankeschön. Das ist etwas, wo man einfach sagen, es ist gut, es lohnt sich, diesen Beruf zu machen.“ (N)<br>• „Ich glaube, wir haben in den verschiedenen Leitlinien, die wir so haben, was die Außerklinik betrifft, eigentlich schon die ganz guten Grundlagen gelegt. Pflegerisch braucht es nicht noch mehr.“ (M)<br>• „Von der Pflege sind sie gut versorgt. Also wir haben den Schlüssel 1 zu 3, der wird eingehalten, das klappt gut.“ (N)<br>• „(…) fast alle (unv.) Patienten (unv.) Kranke. Aber trotzdem, jeder hat Ressourcen, bei jedem kann man was rausholen. (…) Und sage ich, da finde ich einfach immer noch Potential.“ (N)<br>• „Die Tatsache, dass wir unsere Pflegehelfer behalten in der Wohngemeinschaft, obwohl wir sie nicht gegenfinanziert bekommen (…)“ (M)	A positive, committed work attitude and the ability of nurses to recognize and promote each patient's individual resources contribute to needs-based care.<br><br>Providing compassionate and appreciative care and attention to severely ill patients is part of needs-based care, and the patients' gratitude rewards this work.<br><br>The nursing staffing ratio of 1:3 in the outpatient intensive care residential communities is appropriate based on the existing guidelines.<br><br>The employment of additional care assistants, even without refinancing, supports needs-based care.
**Therapists**	
• „Ja, ich sage jetzt einfach mal ganz frech, wir sind so gut aufgestellt, dass wir sowohl logopädisch, ergotherapeutisch, physiologisch, also physiotherapeutisch, bedarfsgerecht versorgen können.“ (N)	Speech-and language therapy, occupational therapy and physiotherapy of good quality and sufficient intensity facilitates needs-based care.
**Physicians**	
• „Was erleichtert die Umsetzung? In meinem Sinn erleichtert die Umsetzung von dieser fachärztlichen Betreuung Telemedizin, weil wir einfach keine Ressourcen haben in Deutschland, (unv.) dass wir so viele Ärzte haben. Deswegen müssen wir über Telemedizin sehr viel arbeiten, was wir auch tun.“ (N)	Telemedicine can facilitate specialist medical care when resources are limited.
**Networking**	
• „Und unsere Hausärztin, die ist auch, also, die kommt, wenn man sie braucht, also die ist sehr gut.“ (N)<br>• „Wir haben in (Ort 1) ein gutes Ärztenetzwerk, sodass wir neurologisch relativ schnell Organisationen, einen Arzt vor Ort haben, der uns helfen kann.“ (N)<br>• „(Ort 3 (AFNT-Team X)) ist für uns wirklich ein kleiner Game Changer jetzt gewesen. Jetzt bezüglich / oder ob das OptiNIV, die die Betreuung über eine wirklich professionelle Institution, die als Ansprechpartner in Notsituationen, aber auch als Ansprechpartner in Bedarfs- und normalen Situationen da war. Da war vor Ort, telefonisch erreichbar, uns wirklich geholfen hat.“ (N)<br>• „Ansonsten, wie (AFNT) sagt, die Überleitung zu den Sozialdiensten. Bewusstsein schärfen ist bei uns toi, toi, toi. Wir haben zwei neurologische Rehakliniken, mit denen wir gut zusammenarbeiten, die gut mit den jeweiligen Sanitätshäusern zusammenarbeiten, und da der Austausch und die Absprache im Vorfeld, Vorverlegung in unsere Wohngemeinschaften schon gut organisiert ist und gut klappt. Tatsächlich der Rollstuhl dann schon im Zimmer ist, wenn der Bewohner da ist. Also das klappt. (…) Da ist diese Zusammenarbeit mit den Sozialdiensten und mit den Sanitätshäusern bei uns gut, toi, toi, toi. Und das ist auch weiterhin wichtig. Und es erleichtert auch die Versorgung, die optimale Versorgung.“ (N)<br>• „Unsere Provider - und ich arbeite mit ganz vielen - bemühen sich sehr, tatsächlich, dass Hilfsmittel rechtzeitig vor Ort sind und helfen tatsächlich und unterstützen auch Angehörige, Betreuer, auch Pflegeversorgung bei Einspruch und Widerspruch.“ (N)	Effective collaboration with GPs and specialists promotes needs-based care.<br><br>An effective, trust-based, and patient-centred cooperation with an outpatient specialist aftercare team is a factor that facilitates the needs-based care.<br><br>The realisation of needs-based care is facilitated by committed and supportive cooperation amongst all stakeholders involved in the provision of medical or technical aids.<br><br>Communication training and coaching to promote constructive communication within the team is a prerequisite for needs-based care.

<table>
<tr><td>„Kommunikationstrainings im Haus. Dass alle einfach miteinander reden können, sich verstehen. Kommunikation (…) - das ist die Grundvoraussetzung für dieses interdisziplinäre Team. (…) Und dazu gehören Kommunikationstrainings von vorne bis hinten und auf allen Ebenen. Kommunikationscoachings (…).“ (M)
„Ein Team, das führen kann, das auch unterscheiden kann zwischen Führung und Management und das in der Lage ist, dieses interdisziplinäre Team auch wirksam zu führen und wirksam werden zu lassen. (…) das Vorleben von einer vernünftigen, konstruktiven Kommunikationsstruktur, weil sonst geht es in die Hecke.“ (M)
„Delegation an Fachpersonal, das ist auch eine Erleichterung. Wenn ich als Atmungstherapeut vor Ort dann die Beatmung optimieren kann, umstellen kann, wenn ich persönlich (unv.) habe, entspricht Blutgasanalyse, transkutane Messung und weiteres.“ (N)</td><td>A team leader in the residential communities who has leadership and management skills and provides optimum support and encouragement to the team also promotes needs-based care.

Nursing staff can be relieved if certain tasks can be delegated to specialists such as respiratory therapists.</td></tr>
<tr><td colspan="2">Financing</td></tr>
<tr><td>„Also, was ich sehr gut finde, sind diese Intensiv-WGs. Weil einfach da Personal ist, das die Patienten bedarfsgerecht versorgen kann. Wie es jetzt bei uns ist, da passt auch der äußere Rahmen, was jetzt zu Hause vielleicht nicht so ist und was, ja, das fördert das auch. Weil es ja finanziell für die Krankenkassen tragbarer ist als wenn die Patienten zu Hause versorgt sind. (…) so finde ich halt, dass die Patienten bei/in den WGs vielleicht doch besser gefördert werden können. Weil wie jetzt mit Physio oder Logopädie, da kommt sie und kann gleich zehn oder elf Patienten machen, spart sich die Fahrten, wie schon die Kollegin gesagt hat. Weil in der Zeit, wo sie da fährt, kann sie natürlich jetzt da elf Patienten nach der Reihe versorgen. Das fördert eigentlich die Umsetzung aus meiner Sicht.“ (N)
„Also, bei uns in der Einrichtung würde ich sagen, ist das organisatorisch schon gut.“ (N)</td><td>Organising the patient care in outpatient intensive care residential communities offers various organisational, financial and personnel advantages compared to care in the home environment.</td></tr>
</table>

Explanations: M - managing; N - nurse; GP - general practitioner.

<u>Thematic field</u>: Implementation of needs-based healthcare <u>Thematic code category:</u> Barriers

Examples of individual statements	Summary statement
Nurses	
• „Also eine zentrale Ansprechperson, die sich dann um die weiteren Inhalte kümmert oder die weiteren Ansprechpartner vermitteln kann. Wenn das nicht möglich ist, wie es häufig in der Fläche nicht möglich ist, dann muss man einfach gucken, dass man sich seine Fachärzte irgendwie herholt und bei Laune hält.“ (N)<br>• „(…) weil man einfach schlecht gutes Personal findet. Weil der Markt einfach leer ist. Es gibt schon wenig Pflegekräfte, das ist anscheinend nicht attraktiv genug, ich weiß es nicht. Und, ja, es ist halt schon eine schwierige Aufgabe, mit solchen Patienten zu arbeiten.“ (N)	In the absence of a central contact person, the nursing staff in the outpatient intensive care residential communities have to deal with the challenging task of finding and retaining medical specialists to care for the patients.<br><br>The recruitment of suitable nursing staff poses a challenge.
Therapists	
• „Und was noch schlecht ist, ist die Logopädie, die sind alle ausgebucht. Wir haben nicht für alle Patienten Logopädie. (…) Bloß als Neurologe das ist ein schwieriges Thema und Logopädie.“ (N)	The need for outpatient speech- and language therapists cannot be fully met.
Physicians	
• „Was ich erlebe, ist, dass Hausärzte oft überfordert damit sind.“ (N)<br>• „Das was bei uns in der WG eben durch den Hausarzt nicht so gegeben ist, weil der ja die ganzen Möglichkeiten gar nicht hat, dass er da eine FEES macht oder sowas.“ (N)<br>• „Ja, wie gesagt, es fehlt tatsächlich fachärztliche Versorgung. (…) Und da kann man schon einen Unterschied erkennen zwischen ländlicher und Versorgung in der Stadt. In der Stadt haben wir tatsächlich viel bessere Versorgung mit den Fachärzten. Auf dem Land finden wir keine Fachärzte. Da ist die erste Behinderung Ressourcen. Wir haben überhaupt keine Ärzte, die zur Verfügung stehen, die überhaupt bereit sind, in die außerklinische Intensivpflege ambulant was zu machen oder hinzufahren und was zu machen.“ (N)	For certain medical issues of neurologically severely affected patients, general practitioners often lack the specialist knowledge and options to provide optimal treatment.<br><br>Outpatient medical specialists who accept the severely affected patients as part of their medical care are difficult to find, especially in rural areas.
Technical aids	
• „Ja, und was noch schwierig ist mit den Krankenkassen. Weil zuerst einmal immer alles, fast alles, abgelehnt wird, was man für einen Patienten braucht, muss man immer kämpfen, dass man die Hilfsmittel oder die Überwachungsgeräte, dass man das genehmigt bekommt.“ (N)<br>• „Die ganzen Versorger jetzt mit entsprechenden Rollstühlen mit entsprechendem Equipment und so weiter. (…) Aber da sehe ich tatsächlich als Behinderungsfaktor die Krankenkasse mit ihren restriktiven Genehmigungsverfahren.“ (N)	Providing patients with medical or technical aids is challenging due to the health insurance companies' restrictive authorisation procedures.
Networking	
• „(…) Das ist das, dass die Patienten häufig schlecht versorgt aus den Kliniken rauskommen. (…) weil die Kliniken mit unseren Patienten einfach massiv überfordert sind. (…) Schon allein weil die Keimlage in den Kliniken viel schlechter ist als bei uns.“ (M)<br>• „Also wenn wir OptiNIV nicht mehr haben, dann behindert uns definitiv die Erreichbarkeit eines Teams, das mit uns Weaning denkt, ohne uns die Patienten aus den Wohngemeinschaften rauszuholen.“ (M)<br>• „Das Einzige, was vielleicht gut wäre, wenn das so frische Fälle sind, wenn da nochmal irgendwie eine intensivere Reha erfolgen würde. Weil das habe ich jetzt bei uns auch noch nicht miterlebt, dass da jemand nochmal auf Reha gekommen ist. Ich weiß nicht, liegt es da dran, dass es wenig Rehaplätze gibt?“ (N)	Necessary acute hospital admissions to local hospitals can be associated with secondary risks, as these hospitals are not specialised for the treatment of neurologically severely affected people.<br><br>Weaning attempts without an interdisciplinary supporting team, as the outpatient specialist aftercare teams in the OptiNiV study, are very unlikely to be feasible in the familiar environment of the outpatient intensive care residential communities.<br><br>The need of subacute patients in outpatient intensive care residential communities for rehabilitation stays is not fully covered.

<table>
<tr><th colspan="2">Financing</th></tr>
<tr><td>

- „(…) Also wir machen deutlich mehr, als wir bezahlt kriegen.“ (M)
- „Eine ziemlich zähe Verhandlungsstrategie rund um die Finanzen.“ (M)
- „Die zähe Verteidigung des Pflegeschlüssels von 1 zu 3.“ (M)
- „Ich weiß, vielleicht gibt es auch die Betrüger, die einfach sagen, wir (…) müssen Patienten haben mit Kanüle (…) und dekanülieren und so weiter.“ (N)
- „Eine echte Herausforderung, die viel behindert, ist, dass wir augenblicklich kein Geld bekommen, wenn die Patienten zum Weaning in der Klinik sind, was / das ist so echt schwierig. Von daher freuen wir uns, wenn wir den Patient bei uns einfach versorgen können.“ (M)
- „Aber wenn ich eine Fachkraft in die Klinik stelle, kriegt / kommt kein Cent zurück. Das kann ich nicht machen. Das geht nicht.“ (M)
- „Und die zweite Behinderung ist: Die, die bereit sind, die Fachärzte, die werden nicht finanziert. Finanzierung ist tatsächlich große Behinderung. (…) Und die Fachärzte sagen auch ganz deutlich oder auch Hausärzte: „Ich kann nicht so viele Hausbesuche machen, weil ich einfach kein Geld bekomme und in der Praxis verdiene ich viel mehr.“ Ganz viele Gespräche hatte ich mit Pneumologen durch (unv.) -Krise, die gesagt haben, wir sind bereit, in die außerklinische Intensivpflege zu gehen und auch Patienten pneumologisch zu behandeln. Aber dafür, dass wir da rausfahren und dann diesen ganzen Bürokratieaufwand auf uns aufnehmen, Potenzialerhebung, Behandlungspläne, Intensivverordnung und so weiter, und dafür, dass man dann am Ende 50, 60€ dafür bekommt, ist einfach zu wenig. Das muss man als große Behinderung / oder ich sehe als große Behinderung.“ (N)
- „Atmungstherapeuten oder Fachpersonal, da ist auch großes Thema Finanzierung. Ich kenne das von unserem Konzern, wir mussten das selber finanzieren. Das heißt, wir werden nicht von den Kassen, von Pflegekassen oder Krankenkassen finanziert, sondern es wird vom Konzern finanziert. Und natürlich können sich es nicht alle Pflegedienste leisten, um so Qualität in der Versorgung aufrechtzuerhalten.“ (N)
- „Die Tatsache, dass wir unsere Pflegehelfer behalten in der Wohngemeinschaft, obwohl wir sie nicht gegenfinanziert bekommen, was ich echt unter aller Kanone finde.“ (M)

</td><td>

Cost negotiations with payers regarding reimbursement for care ratios and services are difficult and complicate patient care. To provide needs-based care, facilities must provide more than they are reimbursed for.

Financial reasons may result in a patient not being decannulated, even though this would be possible from a medical point of view.

Once a patient is admitted to the hospital for weaning from a TC or ventilation, the outpatient intensive care residential communities no longer receive funding. However, they must keep the care bed free for that patient and cannot accompany them to the hospital.

The potential of the patients is often not seen during hospitalisation, and the care of these severely affected patients is typically highly cost-intensive.

Home visits by medical specialists and GP in the residential communities are not adequately refinanced.

Not all nursing services can employ additional respiratory therapists and specialist staff at their own expense unless they are externally funded by health insurance companies.

</td></tr>
</table>

Explanations: M - managing; N - nurse; GP - general practitioner; TC – tracheal cannula.

Thematic field: **Appropriateness of the clinical pathway for the support of needs-based healthcare (medical and organizational aspects)**

Thematic code category: Clinical pathway conceptualization/ positive aspects

• Examples of individual statements	Summary statement
• „Diese einfache Zusammenarbeit von den Therapeuten, Pfleger, Ergotherapeuten, Logopädie, auch Hausarzt. Wir haben einen tollen Hausarzt, auch Neurologen. Nur so kann man schaffen. (…) Und dann sind alle zufrieden, vor allem der Patient." (N)<br>• „Also, wir sind da schon ein Verfechter und unterstützen dieses Konzept. Und ich finde, was Sie jetzt vorgestellt haben, (M1), sehr zielführend. Gerade das Thema Zusammenführen unterschiedlicher Professionen wird doch teilweise eher stiefkindlich gehandhabt. Ist natürlich auch immer abhängig davon, wie die Professionen zusammenarbeiten wollen." (N)<br>• „Und zwar ich sehe diese interdisziplinäre Arbeit sehr wichtig zwischen Therapeuten und Pflegeteam und auch Teamleitungen. Was wir jetzt in der Praxis machen, sind Zielgespräche. Wenn wir einen Patienten aufnehmen, dann gibt es demnächst ein Zielgespräch mit allen Therapeuten und Teamleitungen und Pflegeteam, Bezugspflegekraft dazu. Wo man tatsächlich Ziele festlegt, und dann in der Regel Bezugspflegekraft oder Teamleitung evaluiert diese auch. Ich habe auch SMART Analyse irgendwo gelesen, auch diese realistischen Ziele. Was wollen wir erreichen, wer evaluiert was und so weiter." (M)<br>• „Insgesamt macht der Ansatz so, wie er vorgestellt ist, absolut Sinn; ein Dokumentationsmedium, wo alle reinschreiben. (…) Der Ansatz des Pfades ist richtig." (M)	A positive aspect is the patient-centred collaboration between the different professional groups involved in patient care described in the clinical pathway.<br><br>A common, informative documentation system for all persons involved in patient care, to which everyone has access, is considered useful.

Explanations: M - managing; N - nurse.

Thematic field: **Appropriateness of the clinical pathway for the support of needs-based healthcare (medical and organizational aspects)**

Thematic code category: Clinical pathway conceptualization/ negative aspects

Examples of individual statements	Summary statement
• „Von daher hatten wir immer so ein bisschen Schwierigkeiten, die doppelte Buchführung darzustellen, auch." (M)<br>• „Wobei ich überhaupt nicht bin ist Bürokratie. Es gibt verschiedene Umfragen, wieviel Zeit auf die Dokumentation geht, und wir haben erschreckende Zahlen. Wir haben auch intern im Team das durchgeführt und 50, 40 bis 60 Prozent Arbeitszeit geht an die Dokumentation. Davon müssten wir weg, es müssen wirklich Ressourcen gespart werden und anders eingesetzt werden." (N)	Duplicate or unnecessary documentation should be avoided to save resources.

Explanations: M - managing; N – nurse.

<u>Thematic field:</u> ROFT support for needs-based healthcare <u>Thematic code category:</u> ROFT support/ positive aspects

Examples of individual statements	Summary statement
• „Also, was schon positiv war - die Besuche von der Studie. Also, das war sehr interessant und hat man auch Fragen stellen können, die haben sich die Kanüle und das alles angeschaut. Also das war schon positiv.“ (N)<br>• „Wir haben uns ernst genommen gefühlt, wir hatten einen Gesprächspartner auf Augenhöhe, der auch Ideen hatte und der erreichbar war und dem wir vertraut haben und (…) und das Know How, das sie uns an die Hand gegeben haben. (M)<br>• Und was da sehr gut war, wenn die / als das Team gekommen ist, weil da die FEES gemacht wurde. Das war für uns alle eigentlich ein sehr schöner Moment, weil wir das noch nie gesehen haben und wie der dann da geschluckt hat. Und dann haben die uns Tipps gegeben, dass man den täglich zwei Mal entblockt, damit er einfach, der Schluckreflex angeregt wird und so. Also, das war sehr gut, die Zusammenarbeit.“ (N)<br>• „Und, ja, also, der Kontakt und die Kommunikation, das ist ganz gut, weil /. Da haben wir einmal eine Frage wegen der Kanüle gehabt, also die kümmern sich und haben das dann mit der Logopädie bei ihnen besprochen. Also, das finde ich sehr positiv und finde es halt auch gut, wenn die Patienten einfach so ein Jahr begleitet werden, dass man noch irgendwie was fördern kann und dass man einfach, ja, schon gute medizinische Tipps bekommt einfach. Das was bei uns in der WG eben durch den Hausarzt nicht so gegeben ist, weil der ja die ganzen Möglichkeiten gar nicht hat, dass er da eine FEES macht oder sowas. Also, das habe ich schon sehr gut gefunden.“ (N)<br>• „(…) und haben gesehen die Fortschritte (…)“ (N)<br>• „Das hat den ein oder anderen Krankenhausaufenthalt überflüssig gemacht. Das ist sicherlich ein Argument, das nicht zu unterschätzen ist, gerade bezüglich jetzt gegenüber den Krankenkassen. (…)“ (N)<br>• „Trotzdem kann ich es nachvollziehen, und da hat / haben wir Unterstützung gehabt bei den Ergänzungen. Wir haben keine Möglichkeit, Endoskopie bei uns zu machen. Immer wo wir dann Probleme mit den Kanülen-Umstellungen hatten, mussten wir diese Patienten nicht mehr in die Klinik verlegen, für eine Nachkontrolle konnten das bei uns in der WG machen mit Hilfe von OptiNIV. Das heißt, diese Kanülen- Kontrolle wurde dann in der WG gemacht von OptiNIV, finde ich optimal.“ (N)	It was helpful to be supported by accessible, committed, and reliable contact persons, to receive useful advice for patient care and to have the opportunity to enhance knowledge during the team visits.<br><br>A useful aspect is, that certain diagnostic tests and check-ups could be conducted in the residential community, thus avoiding hospitalisation.

Explanations: ROFT - regional outpatient follow-up team; M - managing; N – nurse.

<u>Thematic field:</u> ROFT support for needs-based healthcare <u>Thematic code category:</u> ROFT support/ negative aspects

Examples of individual statements	Summary statement
• „Wie gesagt, wir haben in der Versorgung schon auch ein Team, was sehr ähnlich OptiNIV Team ist. Und deswegen war das so ein bisschen doppelte Arbeit. Ich bin immer ein Mensch, der auf Ressourcen in der Pflege achtet, deswegen ist immer schade, wenn man doppelt was macht.“ (N)<br>• „Was mir gefehlt hat, ist Absprache von den Terminen. Ich und mein Team und dann auch meine Therapeuten sind sehr gerne dabei, wenn OptiNIV Team in der Versorgung ist. Und dann gab es tatsächlich oft auch Miss-Match bei den Terminen, dass wir überhaupt nicht wussten, dass OptiNIV wieder vor Ort kommt, dann waren wir nicht dabei. Da fehlt dann einfach so diese interdisziplinäre Arbeit vor Ort. (…).“ (N)<br>• „Und damals, das hat mir jetzt persönlich besser gefallen, haben wir immer ein Mal pro Woche die Werte und das per E-Mail geschickt. Und jetzt werden wir täglich angerufen. Das ist ein bisschen, ja, vom Arbeitsablauf her, wenn man gerade in der Pflege ist (…).“ (N)<br>• „Wir haben, das muss ich auch leider erwähnen, auch eine negative Erfahrung, wo wir den Klienten dann tatsächlich stationär hatten. Und das war in meinen Augen keine Optimierung von der Nachversorgung, weil wir danach viele Herausforderungen der Pflege bei uns in der WG hatten.“ (N)<br>• „Was mir dann sehr gefehlt hat, ist, wo Patienten in die Klinik gekommen sind. Wir haben zwei Fälle, zwei sehr negative Fälle. Ein Fall wurde sehr stark geweant in der Klinik, wo wir gar kein Potenzial hatten. Die Meinungen durften sich unterscheiden, aber dann letztendlich ist Patient dann in andere Klinik verstorben. Und das fand ich sehr schade, dass dann in dem / in der Zeit keine Kommunikation stattgefunden hat. Wir konnten uns nicht einbringen in die Versorgung, und das fehlt allgemein, diese Schnittstelle zwischen Klinik und Außerklinik.“ (N)	The additional activities of an external outpatient specialized aftercare team are not efficient if the team in the shared living community already covers all aspects of care.<br><br>Due to a lack of information about planned study visits, interested team members of the outpatient intensive care communities were sometimes unable to participate in the visits and facilitate interdisciplinary collaboration on-site.<br><br>Emails are more suitable for conveying the necessary patient information in the busy day-to-day care setting than the required daily phone calls.<br><br>Outpatient care is not optimal if treatment recommendations lead to a deterioration in the patient's condition and hospital admissions.<br><br>Due to a lack of communication interfaces, the nursing staff of the outpatient intensive care community was unable to exert the desired influence on the care of their patients during hospital stays.

Explanations: ROFT - regional outpatient follow-up team; M - managing; N – nurse.

<u>Thematic field:</u> Additional aspects for needs-based healthcare <u>Thematic code category:</u> Medical aspects

Examples of individual statements	Summary statement
• „Aber trotzdem, jeder hat Ressourcen, bei jedem kann man was rausholen. (…). Mit Kleinigkeiten man muss auch zufrieden sein.“ (N)<br>• „(…) weil das einfach kranke Menschen sind. Dass sie brauchen ab und zu mehr Zuwendung als wir (unv.). Und es ist einfach ein / eine / wenn Pflege einfach mit Patienten reden, sprechen über alle, jede Maßnahme Informationen geben und so weiter.“ (N)<br>• „(…) weil das einfach kranke Menschen sind. Dass sie brauchen ab und zu mehr Zuwendung als wir (…) Und deswegen versuchen wir, diese Zuwendung zu geben, dass sich die Leute wenigstens ein bisschen freuen.“ (N)<br>• „Weil wir möchten halt auch, dass die noch, ja, ein angenehmes Leben haben.“ (N)<br>• „Das ist auch für mich eine der Grundvoraussetzungen, dass der Mensch danach in der Wohngemeinschaft nicht nur Klinikalltag erlebt, sondern auch wirklich wohnen kann.“ (N)<br>• „Und auch, muss ich sagen, ist es so gemeinsam (unv.) wenn da über längere Zeit Pfleger, gleiche Pfleger sind. Langsam bildet sich auch Beziehung zwischen Patienten und Pfleger. (…) die Anfangsphase ist tatsächlich auch die Phase, in der sich Vieles entscheidet, in der viel auf dem Spiel steht. Wo mit Sicherheit am meisten Potenzial herrscht, um da noch mal Stellschrauben anzusetzen oder zu intervenieren.“ (N)<br>• „Und andersrum fehlt mir Interesse von der Klinik. Wenn Patient dann bei uns ist, dass die Klinik sich weiterhin interessiert, wie ist der Lauf von diesen Patienten? (…) Also man müsste die Messung auf Verbesserung abzielen, nicht Null - Eins, sondern graduell in verschiedenen Bereichen. Dann würde da, glaube ich, ganz viel messbar werden, also bei den Patienten, die wir eingeschrieben hatten, definitiv.“ (M)	Every potential for improvement must be utilized and valued to improve the patient's quality of life.<br><br>Prerequisites for needs-based care include creating a positive environment for the patient, a good relationship with the nurse, and communicating all necessary information, such as planned interventions.<br><br>It would be desirable for the treating hospital to be interested in the patient's further development in the outpatient intensive care unit after discharge, as the initial period after discharge is particularly crucial for the patient's further development.<br><br>It is advisable to measure treatment success qualitatively in different areas rather than focussing on goal achievement ‘decannulated’ or ‘weaned’. The achieved improvements by the study patients could thus be shown.

Explanations: M - managing; N – nurse.

Thematic field: **Additional aspects for needs-based healthcare** **Thematic code category:** **Organizational aspects**

Examples of individual statements	Summary statement
• „Natürlich auch wir arbeiten viel mit Familien. Die stehen auch fast jeden Tag täglich hier bei uns in der WG. Wir betreuen die auch die Söhne oder Vater (…)“ (N)<br>• „Man muss ja auch sagen, dass bei uns viele Patienten, die haben Eltern oder Angehörige haben gesagt bekommen, nie wieder in Krankenhaus, wir bleiben hier, bis zu Ende.“ (N)<br>• „Und ich würde es auch gut finden, wenn wir es heute hätten, aber auch allgemein für die Studie auch Stimmen von Teilnehmenden zu hören. Letztendlich geht es um die Patienten, die sich äußern können, dass die auch dann an so einem Interview auch dabei sind, um mitzuteilen, wie war die Studie für die oder wie fanden die dann von außen Zusammenarbeit von Therapien (…).“ (N)<br>• „Und ja (…) es ist vielleicht jetzt auch, wenn es mehr so Sachen gäbe wie die Studie, dass da einfach diese Gruppe mehr Aufmerksamkeit bekommt, das würde das natürlich auch fördern.“ (N)<br>• „Das Einzige, was vielleicht gut wäre, wenn das so frische Fälle sind, wenn da nochmal irgendwie eine intensivere Reha erfolgen würde. (…) Weil die könnten ja doch nochmal mehr fördern als wir jetzt.“ (N)<br>• „Wenn Patient von uns in die Klinik eingewiesen wird, wollen wir weiterhin an der Versorgung teilnehmen.“ (N)<br>• „Ja, was ich so rausgehört habe, ist tatsächlich auch so die vermittelnde Funktion zwischen den Klinik/, (…), vielleicht auch Hausärzten, die ja doch eigentlich die federführende Funktion innehaben sollten und eventuell anderen Fachärzten. Also so diese Vermittlerfunktion.“ (N)<br>• „Und es wäre einfach super, wenn OptiNIV weiterginge, weil ich glaube, es ist versorgungstechnisch ein Gewinn (…)“ (M)<br>• „(…) dass es aus meiner Sicht versorgungstechnisch eine echt gute Sache wäre, wenn OptiNIV in der Form weiterbestehen würde, weil es eine gute Ergänzung ist. Ich glaube, man kann damit auch für die Solidargemeinschaft mittelfristig Einsparungen erreichen. Weil wir, wenn wir OptiNIV allen Patienten zugutekommen lassen könnten, die Weaning-Erfahrung haben und die, die jetzt nicht nur eingeschrieben sind, sondern tatsächlich echt breitflächig. Ich glaube, das würde richtig was reißen. (…) und es kommen mittelfristig Einsparungen raus, wenn man sie richtig misst.“ (M)	Cooperation with relatives is one important aspect in the care of patients in the outpatient intensive care residential communities.<br><br>Patients have expressed the wish to be cared for in the outpatient intensive care residential communities. and with no hospitalisations.<br><br>Participation of study patients in the interviews would have been desirable.<br><br>Further study projects would promote needs-based care in that the group of the severely affected patients in the outpatient intensive care residential communities would receive more attention.<br><br>An intensive interval rehabilitation relatively early after initial neurorehabilitative treatment would be desirable to optimise the progress from the patients in the outpatient intensive care residential communities.<br><br>During hospital stays, the nursing staff of the outpatient intensive care residential community wish to exert further influence on the treatment of patients.<br><br>There should be an organisational body that mediates between the institutions involved (hospital, outpatient intensive care residential community, perhaps GP and medical specialists).<br><br>The continuation of the OptiNIV study would benefit the needs-based care of patients in outpatient intensive care communities and could lead to cost reductions.

Explanations: M - managing; N – nurse; GP - general practitioner.
