# Supplementary material for: Post-discharge “continuum of care” clinical pathway (CP) for persons with severe neuro-disabilities – qualitative research to model needs-based community healthcare, capture the real-life care situation, and assess the appropriateness of the CP's concept with input from community- and hospital-based healthcare professionals
Source: Front Neurol. 2026 May 12;17:1677483. doi: 10.3389/fneur.2026.1677483 (PMC13248884; doi:10.3389/fneur.2026.1677483)
Supplement: Supplementary file 3 [file Data_Sheet_3.pdf]

Supplementary Table 3 (THER-C). Stakeholder- Group: Therapists from the community sector (THER-C).

Individual statements and summary statements for the five thematic fields and ten code categories.

Sequence of presentation (thematic fields):

Description of an appropriate needs-based healthcare

Implementation of needs-based healthcare

Appropriateness of the clinical pathway for the support of needs-based healthcare (medical and organizational aspects)

ROFT support for needs-based healthcare

Additional aspects for needs-based healthcare

Thematic field: Description of an appropriate needs-based healthcare Thematic code category: Content-related aspects

Examples of individual statements	Summary statement
Nurses	
• „Ich bin, glaube ich, die einzige Therapeutin ja hier heute. Und für mich genügt einfach nicht nur satt, sauber und trocken, so wie die Schlagworte sind." (S)	Needs-based care includes more than the minimum of basic nursing care alone.
Therapists	
• „Ja, also als Therapeutin sehe ich natürlich Therapie und Rehabilitation ganz oben (lacht) und finde das eben ganz wichtig, dass die Patienten eben interdisziplinär die Möglichkeit bekommen, sich in allen Bereichen zu verbessern, also sei es jetzt Logopädie, Ergotherapie oder auch Physiotherapie." (S)<br>• „Sondern wirklich auch ganz wichtig eine psychologische Betreuung und ganz wichtig auch eine Diätologin und ernährungsmedizinische Betreuung." (S)	A needs-based care comprises interdisciplinary therapy and rehabilitation, i.e. speech therapy, occupational therapy and physiotherapy to provide the patients with the opportunity to improve in the different areas.<br><br>Psychological consultation, dietary counselling and nutritional medical treatment play an important role in needs-based care.
Technical aids	
• „Es gibt auch Überwachung und Interventionen bei den Patienten. Dass sich ja täglich auch was ändern kann und man dann auch entsprechend handelt und auch medikamentös." (S)<br>• „Und halt einfach die regelmäßigen FEES, also, ja, das würde ich mir einfach sehr wünschen, dass das irgendwie / dass sich da irgendeine Lösung finden würde. Dass man einfach wirklich einen anderen Zugang hat, einen besseren Zugang, dass man einfach wirklich im regelmäßigen Abstand ein bildgebendes Verfahren hat. Weil ich glaube einfach schon, dass man dadurch schneller zu einem positiven Ziel kommt wie jetzt ohne." (S)	Needs-based care includes monitoring and flexible interventions when required.<br><br>The regular fiberoptic endoscopic evaluation of swallowing i.e FEES contributes to needs-based care, as it can accelerate the achievement of goals in the context of dysphagia-management by speech- and language therapy treatment.
Medication	
• „Also ich erlebe das oft, dass so ein Medikamentenplan steht und der dann ewig so bleibt, dass man da einfach auch optimiert auf die Bedürfnisse des Patienten. Das finde ich persönlich schon wichtig." (S)	Needs-based care entails that the medication is tailored to the patient's current needs.

Explanations: S - speech- and language therapist.

Thematic field: Description of an appropriate needs-based healthcare **Thematic code category: Organizational aspects**

Examples of individual statements	Summary statement
Nurses	
• „Und was mir auch persönlich noch wichtig wäre, ist eben Fachpersonal. Leider gibt es ja wie überall Pflegemangel und das merkt man eben auch in der Außerklinischen." (S)	Adequate numbers of qualified care professionals in the residential communities are important for the provision of needs-based care.
Therapists	
• „Ja, und dass es zum Beispiel auch einen Stundenplan gibt, wo eingetragen ist, wenn zum Beispiel Therapeuten kommen (…)." (S)<br>• „Ja, ich könnte es eigentlich ganz kurz sagen. (…) mehr Personal." (S)<br>• „Wenn die, also die Kapazitäten, sind halt leider (…) leider nicht da. Ich bin die einzigste Logopädin bei uns in dieser AIP. Ich bin einmal in der Woche da. Wenn ich Urlaub habe, habe ich gar keinen Ersatz. Das heißt, dann fällt die Therapie komplett aus. Das ist natürlich ein Tropfen auf den heißen Stein, und das wäre, finde ich, gut für die Zukunft, wenn es da irgendein System gäbe, wo natürlich dann auch die Frequenz von der Therapie erhöht werden kann." (S)<br>• „Weil, man bekommt dann meist vom Entlassbrief auch keine große / einen großartigen Diagnosebericht oder Befundbericht. Und das wäre natürlich schon wünschenswert, natürlich dann auch für die Arbeit. Damit man einfach wirklich weiß: „Okay, wo fange ich an mit meiner Therapie, mit welchem Ziel arbeite ich". Und das wäre, finde ich, schon sehr wünschenswert, dass man da einfach, ja, einfach immer mal in regelmäßigen Abständen natürlich dann jemanden hat, mit dem man natürlich erstens mal darüber sprechen kann, dann die Befunde besprechen kann. Und aber natürlich halt auch eine Regelmäßigkeit von überhaupt einem Befund. Das wäre, finde ich, eine bedarfsgerechte Versorgung so medizinisch." (S)	Setting up a timetable in which the appointments of the outpatient therapists are scheduled would be helpful for a good organisation of processes in the residential community.<br><br>Additional therapeutic personnel resources would facilitate needs-based care.<br><br>Outpatient speech- and language therapy treatment should be organised in a way that it can take place with sufficient frequency and on a regular basis for needs-based care.<br><br>The provision of regular reports on medical examinations and an interprofessional exchange of findings and therapy goals support needs-based planning of further speech- and language therapy treatment.
Networking	
• „Ja, und organisatorisch ganz klar halt multidisziplinäres Team, das steht ganz oben. Und dass auch alles koordiniert wird. Also man braucht, denke ich, einfach eine gewisse Struktur (…) und eine Teamleitung (…) und trotzdem ist es ja oft sehr chaotisch und keiner weiß am Ende, wer was zu tun hat. Und da denke ich, müssten sich die Einrichtungen selbst einfach organisatorisch gut aufstellen." (S)<br>• „Ja, und neben diesen ganzen Therapeuten und Pflegeteam spielen natürlich die Angehörigen auch mit im besten Falle, dass man auf die Unterstützung von denen noch zählen kann." (S)<br>• „Auch mit der Delegation ist es nicht immer einfach. Also ich denke, da müsste man sich auch nochmal an einen Tisch setzen und ja, einen Plan finden, wie man das gut umsetzen kann, damit in der Außerklinischen eben mehr Entscheidungsgewalt stattfinden kann." (S)<br>• „Und - jetzt haben wir viele Altenpfleger, die eine Umschulung machen. Und das finde ich auch gut, aber da finde ich, muss auf jeden Fall nochmal genauer hingeschaut werden, dass man das Personal soweit schult, dass sie wirklich fit sind mit TK-Patienten." (S)<br>• „Und ansonsten, da denk ich eben an die eine Einrichtung, die ist da sehr dahinter, die macht sehr viele interne Schulungen, Kompetenzentwicklung, und da merk ich den Unterschied zwischen den beiden Einrichtungen, eben auch bei der Versorgung der Patienten." (S)<br>• „Und ansonsten ist natürlich auch so die Sache des Knowhows, also vom Pflegepersonal und auch von Therapeuten. Und da gibt es natürlich immer welche, die noch am Anfang stehen, und das ist auch absolut legitim. Ich finde es nur wichtig, dass man das transparent auch gestaltet und auch (…) ja, dass zumindest eine Person da ist, die sich mit Trachealkanülen-Management wirklich gut auskennt, weil einfach oft Fehler passieren." (S)<br>• „Dann, ganz wichtig wäre für mich auch, dass die Pflege nicht nur Zeit hat zu pflegen, sondern auch Zeit, um zuzuhören, ja. (…) Oder man muss auch Lust haben, sich eine Viertelstunde und Zeit haben, sich eine Viertelstunde hinzustellen, um einmal zu verstehen, was will der Patient mir jetzt sagen." (S)	The most important factor in the realisation of needs-based care is that patients are cared for by a multidisciplinary team.<br><br>Good organisation, team leadership and management are important factors for successful and efficient teamwork in the residential communities.<br><br>Support from relatives contributes to needs-based care.<br><br>To ensure needs-based care, better coordination and delegation of decision-making powers for the outpatient care structure is necessary.<br><br>Regular training courses and further measures to develop professional expertise provide positive support for needs-based patient care.<br><br>The teams should be organised in such a way as to allow experienced nurses and therapists to support beginners in the field and that there is always a person on site who has specialised knowledge of TC management.

• „Man kann natürlich viel mit der Pflege sprechen, und gerade unsere / der Pflegeleiter, der ist eigentlich eh fast immer da, der kann einen sehr guten Rundumblick geben. Aber es ist natürlich / es wäre was Anderes, wenn einfach ein Team - immer dasselbe Team wäre, und man hat immer seinen wöchentlichen Austausch zum Beispiel.“ (S)	Needs-based care means that even severely affected patients - despite the increased time required - are given the opportunity to express their needs and that nurses and therapists in outpatient care are given the opportunity to implement this time commitment.<br><br>Regular team meetings within a fixed team, for example once a week, would be desirable for needs-based care.
Financing	
• „Ja, ich könnte es eigentlich ganz kurz sagen. Mehr Geld, mehr Personal.“ (S)	Additional financial resources would facilitate needs-based care.

Explanations: S - speech- and language therapist; TC – tracheal cannula.

<u>Thematic field</u>: Implementation of needs-based healthcare <u>Thematic code category:</u> Facilitating aspects

**Examples of individual statements**	**Summary statement**
Nurses	
• „Leider gibt es ja wie überall Pflegemangel und das merkt man eben auch in der Außerklinischen. Und - jetzt haben wir viele Altenpfleger, die eine Umschulung machen.“ (S)<br>• „Was ich jetzt bei uns in der Einrichtung nicht das Gefühl habe ist, dass von der Pflege her, dass die unterbesetzt sind. (…) Also, ich finde, pflegemäßig sind sie sehr gut versorgt.“ (S)	Due to the shortage of nursing staff, many geriatric nurses with retraining are being employed in the residential communities.<br><br>A facilitating factor for needs-based care is the good care-to-patient ratio in the residential community.
Therapists	
• „(…) dann habe ich mit der Pflege geredet, welche Übungen jetzt gut wären, weil, ich muss sagen, da, wo ich gearbeitet habe, das war wirklich toll. Das eine Institut, die haben jeden Tag geübt. Und ich muss schon sagen, hätten die nicht geübt, wäre der Patient nicht so gut geworden. Also das war wirklich unglaublich.“ (S)	Guided speech therapy exercises, which were carried out daily by nursing staff, considerably increased the treatment success.
Physicians	
• „Ich bekomme immer meine Verordnungen vom HNO (…) Also wenn ein neuer Patient gekommen ist, dann hat das Minimum vier Wochen, wenn nicht länger, gedauert, bis ich einmal zu meiner Verordnung gekommen bin und mittlerweile funktioniert das wirklich innerhalb einer Woche. (…) Es kommt eine Verordnung nach der nächsten, und das ist das einzige Bürokratische, was wirklich super funktioniert.“ (S)	If outpatient neurologists are unavailable, treatment prescriptions can also be made by other physicians, such as ENT specialists.
Networking	
• „Also, was fördert die bedarfsgerechte Versorgung, würde ich sagen, aktuell ist definitiv die Zusammenarbeit, also multidisziplinäre Zusammenarbeit, mit allen möglichen Fachgruppen, also sei es Arzt, Pfleger, Therapeuten.“ (S)<br>• „Und ansonsten, da denk ich eben an die eine Einrichtung, die ist da sehr dahinter, die macht sehr viele interne Schulungen, Kompetenzentwicklung, und da merk ich den Unterschied zwischen den beiden Einrichtungen, eben auch bei der Versorgung der Patienten.“ (S)	Needs-based care is facilitated by multidisciplinary cooperation of physicians, nursing staff and therapists.<br><br>Team training and other skills development activities lead to improved quality of needs-based care for patients.
Financing	
• „Ich kann mich jetzt der Kollegin nochmal anschließen mit dem Thema, das ich es auch super finde, überhaupt so dieses ganze Thema mit der WG, dass es überhaupt sowas gibt. Und bei mir ist es halt genau dasselbe - ich mache einen Hausbesuchstag einmal in der Woche. Und das ist auch genau dann der Tag, wo ich dann auch in die WG komme. Weil sich es halt einfach, blöd gesagt, finanziell auch rentiert, weil ich natürlich eben nicht fünf verschiedene Häuser anfahren kann. Könnte ich natürlich schon, aber das ist natürlich dann finanziell jetzt für unsere Praxis dann auch wieder nicht so gern gesehen, logischerweise. Und deswegen finde ich das erstens dafür schon mal ganz gut, weil es halt einfach rentabler ist natürlich. Und ich finde auch, für die Patienten /. Ich finde es wunderschön, weil es ist einfach / es ist wie eine Familie. Das ganze Team hat, finde ich auch, also, man fühlt sich irgendwie wohl, wenn man reinkommt, weil die arbeiten rund um die Uhr miteinander. Die Patienten sind keine Patienten, sondern das sind Mitbewohner, das finde ich auch ganz schön und, genau, also, das finde ich einfach wunderschön.“ (S)	The provision of outpatient intensive care shared living facilities supports needs-based care because patients can live together and several residents can be cared for during therapeutic home visits in a travel- and cost-efficient manner.

Explanations: S - speech- and language therapist.

<u>Thematic field</u>: Implementation of needs-based healthcare <u>Thematic code category:</u> Barriers

**Examples of individual statements**	**Summary statement**
Nurses	
• „Leider gibt es ja wie überall Pflegemangel und das merkt man eben auch in der Außerklinischen.“ (S)<br>• „Und ansonsten ist natürlich auch so die Sache des Knowhows, also vom Pflegepersonal (…) weil einfach oft Fehler passieren. Und ja, das Knowhow einfach fehlt in der außerklinischen Intensivpflege.“ (S)	There is a shortage of nursing staff in the outpatient residential communities.<br><br>A lack of specialised knowledge on the part of nursing staff is a barrier to needs-based care for the patients.
Therapists	
• „Und ansonsten ist natürlich auch so die Sache des Knowhows, also (…) von Therapeuten. (…) weil einfach oft Fehler passieren. Und ja, das Knowhow einfach fehlt in der außerklinischen Intensivpflege.“ (S)<br>• „Viele Patienten bräuchten Therapie und bekommen aber keine Logopädin, also in meinem Bereich, den ich kenne.“ (S)<br>• „Und gerade auch mit TK-Management – ich habe das Gefühl, dass ganz viele so ein bisschen Angst davor haben oder halt einfach so, dass ist ihnen, glaube ich, zu sehr ins Medizinische /. Und deswegen ist natürlich das auch bei uns sehr, sehr schwierig, überhaupt einen Logopäden zu finden.“ (S)<br>• „Wenn die, also die Kapazitäten, sind halt leider, wie das gerade die Kollegin schon gesagt hat, die sind halt leider nicht da. Ich bin die einzige Logopädin bei uns in dieser AIP. Ich bin einmal in der Woche da. Wenn ich Urlaub habe, habe ich gar keinen Ersatz. Das heißt, dann fällt die Therapie komplett aus. Das ist natürlich ein Tropfen auf den heißen Stein (…).“ (S)<br>• „Ich habe leider Gottes auch sehr wenig Austausch mit den Physiotherapeuten oder den Ergotherapeuten, weil die meistens halt auch an anderen Tagen kommen.“ (S)	A lack of specialised knowledge on therapists is a barrier to needs-based care for the patients.<br><br>There is a shortage of speech- and language therapists specializing in in outpatient dysphagia and TC management, meaning many patients cannot receive the treatment they need.<br>Reasons i.e. fear of a lack of expertise and the therapeutic risk.<br><br>The frequency of speech therapy treatment is not sufficient.
Physicians	
• „Weil das Ding ist bei uns, ich bekomme meine Verordnungen nur von einem HNO-Arzt ausgeschrieben. Neurologen haben wir, glaube ich, so gut wie gar nicht bei uns in der Einrichtung. (…) Ich habe noch keine einzige FEES bekommen außerhalb von der Studie, und das ist natürlich dann schon auch schwierig, weil ich habe überhaupt keine Kontrolle von meiner Arbeit. Ich weiß nicht, ob das gut ist, was ich so arbeite mit meinen ganzen Techniken und Manövern und einfach mit meiner Therapie. Ich habe das Gefühl, ich werde so ein kleines bisschen alleingelassen.“ (S)<br>• „Und dann eben, dass auch der Kontakt mit den Ärzten fehlt, dass man regelmäßig seine Schluckdiagnostiken bekommt, und das steht natürlich großartigen Verbesserungen schon sehr im Weg.“ (S)<br>• „Der HNO-Arzt kommt wirklich nur auf mehrfache Anfrage, der muss dann extra herfahren. (…) ist es auch immer schwierig, überhaupt einen Termin zu bekommen, dass überhaupt auch mal ein Arzt dann vor Ort, dass der überhaupt zu einem kommt.“ (S)	A missing contact with the physicians and a lack of prescriptions for swallowing diagnostics to monitor progress and plan further treatment make it difficult to provide needs-based speech- and language therapy.<br><br>Home visits from medical specialists are difficult to obtain for patients in outpatient intensive care.
Technical aids	
• „(…) können nicht reden, und sie wollen trotzdem was äußern. Und dann muss man halt auch mit dieser Kommunikationstafel sich abplagen, dass er einmal irgendwas sagen kann und das geht einfach oft im Trubel dann verloren, wenn es einfach stressig ist.“ (S)	As using the communication board is time-consuming and strenuous, the patients' need to communicate can often not be satisfied in stressful everyday situations.
Medication	
• „Ja, und letztendlich, was ich noch oft schwierig finde oder was ich denke, was wichtig wäre (unv.) Aspekte, also in der Klinik ist es leichter, Medikamente einfach zu geben, in der Außerklinischen wird viel gestrichen, obwohl die Patienten das eigentlich bräuchten.“ (S)	Prescriptions for medication and their adaptations are not as optimal in the outpatient residential communities as they are in hospitals.
Networking	
• „Auch mit der Delegation ist es nicht immer einfach. Also ich denke, da müsste man sich auch nochmal an einen Tisch setzen und ja, einen Plan finden, wie man das gut umsetzen kann, damit in der Außerklinischen eben mehr Entscheidungsgewalt stattfinden kann.“ (S)	The necessary coordination and delegation of authority for the outpatient professions is not sufficient.

• „Also ich kann jetzt nur für die zwei Einrichtungen sprechen, die ich betreue, und da kann ich sagen, dass das meiste Problem eigentlich vom Arzt ausgeht. Also dass der Arzt - ja, wie sag ich das denn, der macht das / also, der delegiert halt fast gar nichts. Der macht so seinen Alleingang. Der möchte auch nicht, dass andere Arztgruppen mit reingezogen werden und das macht die Versorgung sehr, sehr schwer bei uns tatsächlich.“ (S)<br>• „Es gab ein anderes Institut, da war es oft so, wenn ich dann komme, wird genau dann der Patient gewaschen. Oder genau dann wird der Harnbeutel gewechselt, oder genau dann muss inhaliert werden. Also es war oft ganz schwierig, die Therapie zu machen, obwohl ich immer am gleichen Tag immer zur gleichen Zeit komme. Also das war wirklich oft ein bisschen hinderlich, weil ich habe ja nicht mehr Zeit, als ich für diesen einen Patienten habe, ja.“ (S)<br>• „Man (…) würde so viel Sachen über seine normale Therapiezeit noch hinausmachen können, sei es eben Berichte lesen oder sich in Kontakt machen mit den anderen Therapeuten. Aber die Zeit bleibt oftmals einfach gar nicht. Und das ist natürlich schade, weil eben, dann hat man keinen Austausch.“ (S)<br>• „(…) dass überhaupt auch mal ein Arzt dann vor Ort, dass der überhaupt zu einem kommt. Und das ist natürlich dann alles auch ein Zeitfaktor, das geht dann nicht innerhalb von einer Woche. Man muss mehrmals telefonieren, man bekommt dann vielleicht auch die Berichte erst wieder Wochen später. Und das zieht sich dann natürlich alles sehr, sehr in die Länge, und, ja, das ist natürlich sehr schwierig.“ (S)<br>• „(…) dass man oft so das Gefühl hat, die Patienten werden auch in den Kliniken ungern genommen. Dass tatsächlich mehrmals Kliniken die Patienten so bisschen abweisen (…)“ (S)	Poor coordination, delegation and teamwork on the part of the physician providing medical care in two residential communities makes it difficult to deliver needs-based care to the patients.<br><br>Overlaps between therapy sessions and care activities despite fixed and regular therapy appointments make it difficult to provide needs-based (speech- und language therapy) care.<br><br>There are no time resources for activities that go beyond direct (speech- and language) therapy work with the patient, e.g. for exchanging information with other therapists or reading medical reports.<br><br>It is a lengthy process from organising an appointment with the physician in the residential community to being sent the medical results.<br><br>At some hospitals, attempts are made to avoid admitting the severely neurologically affected patients from outpatient intensive care.
Financing	
• „Und dann Thema Kosten, hatte ich ja auch schon gesagt, erlebe ich tagtäglich, dass manche Sachen nicht bestellt werden können. Das fängt schon bei Absaugkathetern an und endet bei der Kompresse. Dass es einfach immer nie die Möglichkeit gibt, irgendwelche Sachen zu bestellen, weil das nicht bezahlt wird, also von Seiten der Krankenkasse, ist auf jeden Fall auch noch eine Notwendigkeit da zur Verbesserung.“ (S)<br>• „Das Einzige, was mir jetzt noch eingefallen wäre, ist, dass es ja auch noch andere Berufsgruppen gibt, von denen die Patienten profitieren können. Also, es gibt ja nicht nur Ergo, Logo, Physio, es gibt ja auch noch Osteopathen und Heilpraktiker. Und, ja, das ist natürlich im Budget nicht drin bei den gesetzlich Versicherten.“ (S)<br>• „(…) und dass jetzt ein Patient in einer Klinik in der nächstgrößeren Stadt versucht wird, aufgenommen zu werden, weil die anderen Kliniken das irgendwie nicht machen. Und das ist natürlich schon auch schwierig, weil erstens ist es dann natürlich auch wieder mit einem finanziellen Aspekt verbunden. Ich brauche einen Transport, das stresst meinen Patienten, wenn der aus seiner natürlichen Umgebung von der WG rauskommt. Also, das ist sowohl finanziell natürlich wiederum ein Aufwand, aber natürlich auch für den Patienten. Und, ja, das finde ich natürlich sehr schade, dass einfach die Versorgung so nicht gegeben ist, wie es halt einfach vor Ort sein könnte.“ (S)	Due to the lack of cost coverage by health insurance companies, necessary nursing consumables cannot be reordered in sufficient quantities.<br><br>One barrier to needs-based care is that the costs of certain treatments (such as osteopathy and alternative medical treatments) are not covered by statutory health insurances.<br><br>Due to limited medical care options in smaller community hospitals hospital treatments may be required in larger urban hospitals, which places a greater burden on the patient due to longer transport distances and higher costs.

Explanations: S - speech- and language therapist; TC – tracheal cannula.

Thematic field: **Appropriateness of the clinical pathway for the support of needs-based healthcare (medical and organizational aspects)**

Thematic code category: **Clinical pathway conceptualization/ positive aspects**

Examples of individual statements	Summary statement
• „Ich finde es eigentlich auch super, wenn das so einheitlich und kompakt irgendwie eine Mappe ist, und man sieht sofort von allen Disziplinen, was ist der aktuelle IST-Zustand, was ist das Ziel. (…) Bei uns sind zumindest die therapeutischen Berufe doch alle in einem Ordner, also das ist sehr Old School, also mit handschriftlichem Verlauf. (…) Jeder Therapeut hat seinen eigenen Zettel, wo man auch nachschauen könnte. Da wäre aber natürlich dann auch wieder, was wir vorhin schon gemeint gehabt haben, also, natürlich dann auch wieder dieser zeitliche Faktor. Muss ich recht lange in dem Ordner dann rumblättern, habe ich alles gleich auf einen Blick beieinander. Aber das Ziel an sich finde ich schon sehr gut, dass man das wirklich alles kompakt auf einen Blick hat, weil es halt einfach dann schon Zeit sparen würde auf jeden Fall.“ (S)<br>• „Idee mit diesem Entlassbrief, dass man dann zum Beispiel auch dann die Nummern dabei hat /. Ich weiß nicht, ob ich es richtig verstanden habe, dass das dann quasi die Nummern sind von der Klinik, vom Akuthaus, wo die dann entlassen werden, weil das, weiß ich nicht, wie das umsetzbar wäre. Ich meine, die Idee ist super, weil man eben dann einen Ansprechpartner hat. Weil, wenn ich natürlich erst in der Klinik mich durchtelefonieren muss, bis ich dann wirklich bei demjenigen ankomme, ist das natürlich dann auch wieder ganz viel Zeit, was verloren geht.“ (S)<br>• „Und auch mit diesen Teamkonferenzen fände ich auch gut, wenn man das auch wirklich vielleicht sogar mit – so, wie jetzt hier – mit so einem Zoom-Meeting machen könnte.“ (S)	A documentation system should present the status and objectives of the patient in a clear, compact, and standardised way for all professional groups involved in patient care in a folder, thus making it possible to obtain the information briefly in a time-saving manner.<br><br>Providing contact information of other professionals involved in care as part of the documentation would be useful.<br><br>Team conferences could be held online via video-calling.

Explanations: S - speech- and language therapist.

Thematic field: **Appropriateness of the clinical pathway for the support of needs-based healthcare (medical and organizational aspects)**

Thematic code category: **Clinical pathway conceptualization/ negative aspects**

Examples of individual statements	Summary statement
• „Aber, ja, einfach die Kommunikation zwischen den Therapeuten, da scheitert es einfach oft, oder das ist halt einfach oft sc hwierig.“ (S)<br>• „Ja. Mich hat es furchtbar genervt, muss ich ehrlich sagen, weil es für mich eine dreifache Dokumentation war. Ich habe meine eigene Dokumentation, wo ich auch immer reinschreibe, was die Pflege mir ja sagt, wie es dem Patienten geht und ob die Übungen funktioniert haben und wie es mit Essen geht und wie es mit der Kanüle geht, und das Ganze habe ich mir aufgeschrieben. Dann habe ich die Therapie gemacht, dann habe ich mit der Pflege geredet, welche Übungen jetzt gut wären (…) So, dann habe ich denen das erklärt, was die nächste Übung ist. Dann habe ich die Übung auch aufgeschrieben, weil es musste ja korrekt weitergegeben werden, dann war meine Dokumentation fertig. Dann musste ich im Patientenordner dokumentieren und dann musste ich noch hier dokumentieren, und das war wirklich / ich dokumentiere und dokumentiere, und irgendwie war mir dann immer die Therapiezeit zu kurz (…).“ (S)	Communication between therapeutic professions can be improved through written documentation. However, a uniform documentation system would need to be implemented to avoid duplication of documentation.

Explanations: S - speech- and language therapist.

<u>Thematic field:</u> ROFT support for needs-based healthcare <u>Thematic code category:</u> ROFT support/ positive aspects

Examples of individual statements	Summary statement
• „Ich fand es sehr gut, dass, dadurch, dass der Arzt sich in der Einrichtung eben immer sehr quer gestellt hat, war das sehr gut, dass eben von der (Ort 4 (AFNT-Team X)) -Seite da interveniert wurde und der Arzt ein bisschen Druck bekommen hat (lacht) und dann endlich was passiert ist.“ (S)<br>• „Ich fand das auch mit der Doku gut. Ich habe mich da auch darangehalten, obwohl da noch die doppelte Doku quasi erfolgen musste.“ (S)<br>• „(…) also Pflegepersonal war eher sehr genervt (lacht), tatsächlich. Die sind froh teilweise, dass das jetzt ENDLICH vorbei ist, weil ja so oft angerufen wurde. Also, für Viele war das sehr zeitintensiv, was ich sehr schade finde, weil es ja auch zum Wohl der Patienten war. Ja. Also, grundsätzlich fand ich das einfach gut, dass immer wieder nachgefragt wurde.“ (S)<br>• „Ja, ich hatte immer Glück mit den Kolleginnen vor Ort, wo ich mich dann rück-, also wo ich Rücksprache halten konnte. Wir hatten einen sehr psychisch auffälligen Patienten. Und da war es einfach interessant, wie war er vor Ort in der Klinik und wie war er dann bei uns. Und was waren die Fortschritte und gibt es irgendwelche Tipps, was sie gemacht haben, was ich machen könnte, und von dem war die Zusammenarbeit sehr, sehr angenehm.“ (S)<br>• „Und ich glaube schon, dass, dank der Studie oder dank den regelmäßigen FEESen, ein Patient definitiv schneller zur Dekanülierung gekommen ist wie wenn es jetzt nicht mit der Studie gewesen wäre. (…) Und nur durch diese regelmäßige Kontrolle oder überhaupt, dass ich einfach mal auch einen Einblick gehabt habe auf diese ganzen Strukturen und so weiter, ist das natürlich dann viel, viel schneller zur Dekanülierung gekommen. Weil ich hätte mit Sicherheit die Entblockungszeiten nicht in diesen / in dieser Schnelligkeit quasi erhöht, weil ich mir einfach unsicher war ohne diese bildgebenden Verfahren.“ (S)<br>• „Auch einfach auch immer diesen Kontakt zum Neurologen nochmal zu haben, auch mit einer anderen Logopädin. Dass man einfach auch nochmal den Austausch zwischen den / also, zwischen Logopädinnen hat, das finde ich auch sehr wichtig.“ (S)	Medical care has been improved in the residential community through the work of the outpatient aftercare study team.<br><br>The documentation for the study was positive, despite the need for double documentation during this period.<br><br>The regular phone calls and follow-up enquiries from the outpatient aftercare study team constitute a positive aspect.<br><br>The collaboration with the outpatient aftercare study team was very pleasant, interesting, and helpful.<br><br>The study's regular swallowing examinations provided more certainty and opportunities for the planning and execution of the speech therapy treatment, which could accelerate decannulation.<br><br>The opportunity for interdisciplinary contact and exchange with neurologists and speech therapists during the study was helpful.

Explanations: ROFT - regional outpatient follow-up team; S - speech- and language therapist.

<u>Thematic field:</u> ROFT support for needs-based healthcare <u>Thematic code category:</u> ROFT support/ negative aspects

Examples of individual statements	Summary statement
• „Aber ich habe auch Gegenteiliges gehört, also Pflegepersonal war eher sehr genervt (…), tatsächlich. Die sind froh teilweise, dass das jetzt ENDLICH vorbei ist, weil ja so oft angerufen wurde. Also, für Viele war das sehr zeitintensiv, was ich sehr schade finde, weil es ja auch zum Wohl der Patienten war.“ (S)<br>• „Was nicht so gut war, war - bei einer Patientin wurde eine Schluckuntersuchung durchgeführt, eine FEES. Und das war organisatorisch und auch inhaltlich nicht wirklich gut, muss ich ehrlich sagen, vor allem, weil wir ja auch ein FEES-Gerät haben, das wir vor Ort reinigen können, und da hätte man da wiederum bisschen besser kommuniziert, hätte man sich das auch vielleicht sparen können. Und letztendlich hat die FEES auch gar nicht funktioniert, und das fand ich irgendwie schade. Da wurde dann so ein Alleingang gemacht, und das fand ich / das wollte ich auch noch rückmelden. Ja. Dass diese FEES, die von der Klinik aus kam, nicht gut war vom Setting. Und hätte man besser machen können, ja.“ (S)	According to information from the nursing staff, the study and the associated daily calls from the outpatient aftercare study team were sometimes perceived by the nursing staff as very time-consuming and annoying.<br><br>There was a negative experience with a fiberoptic endoscopic evaluation (FEES) of swallowing of a patient, planned and carried out by the outpatient aftercare study team, where the arrangements and organisation with the outpatient intensive care team were not optimal.

Explanations: ROFT - regional outpatient follow-up team; S - speech- and language therapist.

<u>Thematic field:</u> Additional aspects for needs-based healthcare

<u>Thematic code category:</u> Medical aspects

Examples of individual statements	Summary statement
• „Das Einzige, was mir jetzt noch eingefallen wäre, ist, dass es ja auch noch andere Berufsgruppen gibt, von denen die Patienten profitieren können. Also, es gibt ja nicht nur Ergo, Logo, Physio, es gibt ja auch noch Osteopathen und Heilpraktiker.“ (S)<br>• „(…) oder, ja, dann einfach nur mal massiert werden, ohne jetzt diesen physiotherapeutischen Hintergrund. Ich weiß nicht, ob das jetzt hier so reinpasst, aber das sind auch Dinge die, finde ich, das Leben der Patienten verbessern können oder verschönern können. Vor allem, wenn sie palliativ sind.“ (S)	Patients can also benefit from the work of other therapeutic professionals such as osteopaths, alternative practitioners, or massage therapists.

Explanations: speech- and language therapist.

<u>Thematic field:</u> Additional aspects for needs-based healthcare <u>Thematic code category:</u> Organizational aspects

Examples of individual statements	Summary statement
• „(…) erlebe ich tagtäglich, dass manche Sachen nicht bestellt werden können. Das fängt schon bei Absaugkathetern an und endet bei der Kompresse. Dass es einfach immer nie die Möglichkeit gibt, irgendwelche Sachen zu bestellen, weil das nicht bezahlt wird, also von Seiten der Krankenkasse, ist auf jeden Fall auch noch eine Notwendigkeit da zur Verbesserung. Ja.“ (S)<br>• „Dass sie vielleicht auch noch mehr Therapie bekommen (…)“ (S)<br>• „Dass sie vielleicht auch noch mehr Therapie bekommen oder von zwei verschiedenen Therapeuten (…)“ (S)	Cost coverage by health insurance companies and the associated possibility of supplying medical and care aids is severely limited and therefore in need of improvement.<br><br>Within the context of needs-based care, patients could receive more treatments from therapists with diverse professional backgrounds.

Explanations: speech- and language therapist.
