# Supplementary material for: Post-discharge “continuum of care” clinical pathway (CP) for persons with severe neuro-disabilities – qualitative research to model needs-based community healthcare, capture the real-life care situation, and assess the appropriateness of the CP's concept with input from community- and hospital-based healthcare professionals
Source: Front Neurol. 2026 May 12;17:1677483. doi: 10.3389/fneur.2026.1677483 (PMC13248884; doi:10.3389/fneur.2026.1677483)
Supplement: Supplementary file 4 [file Data_Sheet_4.pdf]

Supplementary Table 4 (ROFT). Stakeholder- Group: Regional outpatient follow-up team (ROFT).

Individual statements and summary statements for the five thematic fields and ten code categories.

Sequence of presentation (thematic fields):

Description of an appropriate needs-based healthcare

Implementation of needs-based healthcare

Appropriateness of the clinical pathway for the support of needs-based healthcare (medical and organizational aspects)

ROFT support for needs-based healthcare

Additional aspects for needs-based healthcare

<u>Thematic field</u>: Description of an appropriate needs-based healthcare <u>Thematic code category:</u> Content-related aspects

**Examples of individual statements**	**Summary statement**
Therapists	
• „Ja, also mir fehlt jetzt nur noch (lacht) natürlich die adäquate Logopädie, aber auch Physiotherapie und Ergotherapie. Also dass das auch noch adäquat eingebunden wird (…) von der Betreuung her, ja." (P)<br>• „Wir bräuchten ein Therapeutenteam, welches sich ebenfalls diesen Patienten widmen kann (…)" (O)	Needs-based care involves speech- and language therapy, physiotherapy, and occupational therapy treatment.
Physicians	
• „Das heißt, der Patient bräuchte einerseits die ärztliche Betreuung, die sich diesem Thema auch widmen kann." (O)	Needs-based care involves adequate care by physicians.

Explanations: O - occupational therapist.

Thematic field: Description of an appropriate needs-based healthcare Thematic code category: Organizational aspects

**Examples of individual statements**	**Summary statement**
Nurses	
• „Also von meiner Seite, wenn man die bedarfsgerechte Versorgung im Idealfall ansieht, sollte sie ja sich letztendlich an den Krankheitsbildern der Patienten orientieren. Und wir sprechen ja hier wahrscheinlich vor allen Dingen von neurologisch und pneumologisch erkrankten Patienten. Das heißt, wir müssten quasi eine Versorgung außerklinisch herstellen oder etablieren, die diesen Patienten gerecht werden kann.“ (O)<br>• „Ebenso sieht es aus meiner Erfahrung, sieht es meiner Meinung nach so aus, dass die Pflegenden, die dort arbeiten, sich sukzessiv durch Fortbildung bzw. auch durch einen festen Wissensstand diesen Patienten widmen können müssen und es ungünstig ist, wenn es quasi nur kurze Fortbildungen gibt, die dann aber letztendlich, ja, nur in einer Basisqualifikation münden und danach nicht mehr weiter ausgebaut werden.“ (O)<br>• „Organisatorisch kann ich da zusammenfassen, dass ich der Meinung bin, dass eigentlich alles aus einer Hand stattfinden muss. (…) Ich glaube, es wäre günstig, wenn man ein ähnliches Konstrukt baut wie die neurologische Frühreha. Dass quasi die Organisation der außerklinischen Intensivpflege nicht nur die Pflege umfasst, sondern auch den Arzt und die Therapie verpflichtend umfasst, sodass es quasi alles aus einer Hand für den Patienten die Leistung gegeben wird, sodass auch der Überblick gewahrt bleibt, dass jeder Patient quasi zu gleichen Maßen die für ihn bedarfsgerechte Therapie erhält.“ (O)<br>• „Wir bräuchten ein Therapeutenteam (…) das quasi direkt durch diese betreffenden Ärzte delegiert wird.“ (O)<br>• „Ich glaube, es sollte insgesamt ein Non-Profit Unternehmen sein, die Pflege dieser betroffenen Patienten.“ (O)<br>• „Und man ein klares Zeichen setzen muss, dass eine / nur die Kanüle als solche nicht die Grundlage einer Pflegebedürftigkeit ist (…) Und die AKI hätte viel mehr Sicherheit im Handling der Patienten und könnte vielleicht auch potenzielle Dekanülierungen vorantreiben, wenn der Patient danach auch eine Grundlage hätte, finanziert zu werden und in der Einrichtung bleiben DÜRFTE. Das wäre im Reha-Verlauf, also dem medizinischen Verlauf, äußerst dienlich.“ (O)	I Nursing care should be organized in such a way that it is tailored to the specific requirements of these neurologically and pneumological severely ill patients and that it meets the needs of these patients.<br><br>In the area of nursing care, it is important to continuously expand specialist knowledge by participating in further training programmes to be able to provide good care for severely affected patients.<br><br>Residential intensive care should be organized in such a way that it includes the professional groups of nursing as well as physicians and therapists on a mandatory basis and that the services for patients are organized centrally and delegated by the medical staff.<br><br>Care for the patients with severe neurological disorders should be provided by non-profit organizations.<br><br>The need for care should not be defined by the presence of a tracheal cannula and after decannulation or mechanical ventilation and after weaning, there should be a financing option for continued care of these severely affected patients in the outpatient intensive care residential community.
Therapists	
• „Wir bräuchten ein Therapeutenteam, welches sich ebenfalls diesen Patienten widmen kann, was Fähigkeiten und Fortbildungen angeht (…)“(O)<br>• „Ich (…) würde nochmal auch in Richtung Therapie denken, (…) also einmal diesen Zugang zu Therapien, aber auch in ausreichendem Zeitumfang.“ (S)<br>• „Ich denke auch, dass, ja, dass die Etablierung neuer Berufsgruppen wie zum Beispiel dieser Schnittstellenberufe Atmungstherapie als Zwischenstück zwischen Pflege, Therapie und Arzt mit vielleicht auch delegierbaren Befugnissen hier eine Entlastung bringen kann, gerade im Feld der AKI.“ (O)	A team of therapists is needed that has the necessary knowledge and training to work with this group of patients with adequate time frames.<br><br>New professions such as respiratory therapists, perhaps with delegation authorization offer the potential to effectively support the difficult care situation in residential intensive nursing care.
Physicians	
• „Das heißt, der Patient bräuchte einerseits die ärztliche Betreuung, die sich diesem Thema auch widmen kann. Wir bräuchten also eben keinen Hausarzt, sondern zumindest Mediziner, egal, ob neurologisch oder internistisch, die auch eine Intensiv-Zusatzqualifikation haben und diese Patienten behandeln dürfen und können, vollumfänglich.“ (O)<br>• „Und zuletzt auch die Diagnostikelemente, also dieser Zugang zu den betreuenden Ärzten oder Fachdisziplinen, aber auch Diagnostik-Mitteln, FEES, Tracheoskopie ist da ein großer Bereich (…)“ (S)	Medical treatment should be provided by internal medicine or neurology consultants with specialist qualifications in intensive care medicine who have experience with this patient group and are able to carry out diagnostic interventions.
Technical aids	
• „Was auch noch manchmal die Umsetzung einer guten Weiterbetreuung verhindert ist, dass zum Teil die Hilfsmittel eben nicht adäquat vorhanden sind. Und da müssen sich vielleicht auch die neurologischen Rehakliniken ein bisschen zur Hand nehmen. (…) Also das müssen wir	The supply of medical and technical aids should be organized by the discharging rehabilitation center in a timely manner, supported

vielleicht auch noch den Kliniken an die Hand geben, dass wirklich die Hilfsmittelversorgung gleich nach der Entlassung, die muss einfach stimmen, die muss eigentlich sofort schon da sein. Gut, vielleicht das ein oder andere Hilfsmittel, was man erst später benötigt, wie zum Beispiel manchmal ein Kommunikationsgerät, das braucht man vielleicht am Anfang noch nicht, das ist erst später dann sinnvoll. Aber sowas wie Rollstühle sollte sowas sein wie ein Bett. Dass das, dass das in der Intensiv-WG vorhanden ist.“ (P)	by the team in the selected residential intensive nursing care home to ensure that all necessary assistive devices are already available on discharge from the rehabilitation center.
Networking	
• „Des Weiteren denke ich, dass es günstig wäre, gewisse Mindeststandards sowohl für Ausbildungen als auch für den Austausch eines Teams oder eine Team- / ja, überhaupt der Aufbau eines Teams eigentlich verpflichtend sein sollte, dass man sich austauscht und Ziele für den Patienten formuliert.“ (O)<br>• „Und was - denke ich - ganz wichtig wäre, dass man gerade in der Anfangszeit, wenn der Patient frisch in die Intensiv-WG kommt, dass man da einen sehr engen Kontakt hat. (…) Von der vorhergehenden Rehaklinik oder halt so ein Team und später halt braucht es vielleicht nicht mehr ganz so eng sein.“ (P)	Minimum standards should be set for professional training and for the work in teams, which should include the topics of collaboration, communication, and goal setting.<br><br>In the initial period after the patient's admission from the rehabilitation clinic to the outpatient intensive care residential community, close support from an outpatient aftercare specialist team, for example provided by the rehabilitation clinic, would be an important factor for needs-based care.
Financing	
• „Manchmal frage ich mich, ob vielleicht auch eine – ich sag jetzt mal – bezahlte Zeit für diese Dinge relevant ist, also für interdisziplinären Austausch mit dem Hausarzt, mit dem Neurologen, mit / vielleicht auch für eine Schulung vom interdisziplinären Team. (…) Also, so eine ausführliche Befassung mit dem Fall, denke ich mir manchmal, das wäre auch spannend, wenn man da sozusagen nicht ins Minus in seinen Stunden oder in seinem Gehalt geht, nur weil man sich jetzt versucht, gut auf den Patienten vorzubereiten und das Sinnvolle zu tun.“ (S)	Cost coverage for the time required for interdisciplinary exchange, intensive preparation for a patient and team training would be a prerequisite for need-based care.

Explanations: O - occupational therapist; P - physician; S - speech- and language therapist.

Thematic field: Implementation of needs-based healthcare Thematic code category: Facilitating aspects

Examples of individual statements	Summary statement
Nurses	
• „Genau, was ich auch gut finde ist, dass es, auch wenn die Fortbildung leider nicht kontinuierlich gewährleistet ist, dass wir dennoch die Möglichkeit haben in der AKI, und ich glaube, das wird da mehr betrieben teilweise sogar als im Krankenhaus, dass man Menschen, die zum Beispiel einen Pflegeberuf, Pflegehelfer oder Ähnliches gelernt haben, die Möglichkeit gibt, sich hier weiter zu differenzieren und vielleicht auch weiter auszubilden." (O)<br>• „Möchte eine Sache noch hinzufügen – engagierte Menschen (…) engagierte Mitarbeitende. Ich glaube, die sind tatsächlich der Schlüssel dafür, für eine erfolgreiche und eine bedarfsgerechte Versorgung." (S)	The implementation of needs-based care is supported by the existing training and further education opportunities for the nursing staff in the outpatient intensive care residential communities.<br><br>Dedicated people (employees in the outpatient intensive care residential communities) are the key to needs-based care.
Therapists	
• „Möchte eine Sache noch hinzufügen – engagierte Menschen. Engagierte Therapeuten (…). Ich glaube, die sind tatsächlich der Schlüssel dafür, für eine erfolgreiche und eine bedarfsgerechte Versorgung." (S)	Dedicated people (therapists) are the key to needs-based care.
Networking	
• „Also was die Umsetzung erleichtert ist, denke ich, wenn die Intensiv-WGs, wie es meistens wohl der Fall ist, auch den Patienten sich schon vorher in der Reha anschauen und man alle Probleme bespricht." (P)	The nursing staff in the outpatient intensive care residential communities often become acquainted with the patient during inpatient rehabilitation already and use the opportunity to exchange information with the carers in the clinic, which promotes needs-based care.
Financing	
• „Also ich glaube, gerade wenn man international das sich anschaut, ist es vor allen Dingen die gute Finanzierungslage, die wir aktuell eigentlich haben, (hustet) wenn auch gleich sie vielleicht ungünstig verteilt ist beziehungsweise nicht auf / also, da sie gewinnbringend oft sein muss. Also wir / die / im Prinzip ist es ja schon so, dass man als Patient in Deutschland mit so einer schweren Erkrankung darauf hoffen darf, dass man eigentlich eine finanzierbare Unterbringung geniest mit einem relativ guten Pflegeschlüssel. (…) Und, ja, ansonsten glaube ich auch, dass insgesamt unser / ja, dass das Gesundheitssystem, ja, vor allem im finanziellen Bereich, wie gesagt, die Versorgungslage ganz gut ist, ne, also auch, was materielle Dinge dann auch angeht, haben wir eigentlich einen relativ guten Standard hier in Deutschland." (O)	There is an overall good financial situation for the care of the severely neurologically affected patients in Germany with a satisfying standard in terms of accommodation and equipment.

Explanations: O - occupational therapist; P - physician; S - speech- and language therapist

.

<u>Thematic field</u>: Implementation of needs-based healthcare <u>Thematic code category:</u> Barriers

Examples of individual statements	Summary statement
Nurses	
• „Genauso wenig kann ich erwarten, dass eine Pflegefachperson, die vielleicht eine Grundlagen-/, oder die ihre Ausbildung im geriatrischen oder pädiatrischen Bereich absolviert hat, nun mit erwachsenen neurologisch schwerstbetroffenen Patienten mit Beatmung arbeitet, ohne dass sie geeignete Supervision oder Ausbildungsmöglichkeiten erhält, die über die jetzigen einwöchigen und zweiwöchigen Fachkurse hinausgehen." (O)<br>• „Also auch wenn wir darüber sprechen es gibt (…) zu wenig Pflegekräfte. (…) Wir sind perspektivisch immer mehr auch in dem Arbeitsmarkt, wo es einfach sehr viel mehr Stellen gibt als Menschen, die auf diesen Stellen arbeiten können. (…) Aber ich kann total verstehen, dass das ein Faktor ist, der uns als Gesellschaft betrifft." (S)<br>• „Ich glaube, auch das ist eine finanzielle Frage. (…) Wer entscheidet sich bewusst für einen Pflege- (…) beruf, der/wo man nicht die goldene Nase verdient (…) Und auch die Frage, welche Arbeitsbedingungen sind das." (S)	Insufficient qualification of nurses regarding the treatment and care of ventilated patients hinders an optimal development of the patients.<br><br>There is a shortage of nursing staff - a situation where more job positions are available than can be filled (due to income and working conditions), being relevant to society.
Therapists	
• „Ich kann nicht erwarten von einem Ergotherapeuten, der frisch von der Schule kommt, dass er einen schwerstbetroffenen beatmeten Patienten in einer AKI behandle." (O)<br>• „Also auch wenn wir darüber sprechen es gibt zu wenig Therapeut*innen (…) Wir sind perspektivisch immer mehr auch in dem Arbeitsmarkt, wo es einfach sehr viel mehr Stellen gibt als Menschen, die auf diesen Stellen arbeiten können. (…) Aber ich kann total verstehen, dass das ein Faktor ist, der uns als Gesellschaft betrifft." (S)<br>• „Ich glaube, auch das ist eine finanzielle Frage. (…) Wer entscheidet sich bewusst für (…) einen Therapieberuf, der/wo man nicht die goldene Nase verdient (…) Und auch die Frage, welche Arbeitsbedingungen sind das." (S)<br>• „Und genau (…) es gibt auch wenig Leute, die sich das zutrauen, auch weil das kein fester Teil der Ausbildung ist. Es ist nicht Teil der Prüfungsordnung, der Logopädischen. Das heißt, man könnte theoretisch eine logopädische Ausbildung machen, ohne ein was darüber gehört zu haben, wie man Trachealkanülen behandelt." (S)<br>• „Mir ist noch eingefallen, also, wir hatten jetzt schon die Therapeuten, die eigentlich nicht die Ausbildung jetzt per Haus aus haben, so, das zu machen und sich (…) potentiell mit dem Weg in die Praxis (…), ja, gegen ein klinisches Setting und die schwerstbetroffen Patienten POTENTIELL entscheiden. (…) Weil die Leute ja sich irgendwie auch bewusst gegen / vielleicht auch bewusst gegen diese intensivmedizinischen Patienten entschieden haben (…)." (S)<br>• „Auch das ist abrechnungstechnisch nicht ganz so einfach. Aber auch so die Frage - es sind wahnsinnig komplexe Fälle, mit denen wir arbeiten, wir haben Patient*innen, die haben 80 Arztbriefe, bevor sie bei uns in die Therapie kommen - wann soll ich die lesen?" (S)	Insufficient qualification of therapists regarding the treatment and care of ventilated patients hinders an optimal development of the patients.<br><br>There is a shortage of therapists - a situation where more job positions are available than can be filled (due to income and working conditions), being relevant to society.<br><br>The basic professional training for therapists does not (sufficiently) qualify to work with patients with TC, so that many health care workers do not have the confidence to work with the severely affected patients.<br><br>Time required to read the extensive medical reports in preparation for the therapeutic treatment of a new patient with a complex medical background is not remunerated separately.
Physicians	
• „Es kann nicht sein / Ich habe jetzt wieder das Problem mit einem Hausarzt gehabt, der leider sich mit Beatmung gar nicht auskennt." (O)<br>• „Es kann nicht sein / Ich habe jetzt wieder das Problem mit einem Hausarzt gehabt, der leider sich mit Beatmung gar nicht auskennt. Der aber gleichsam der behandelnde Arzt des Patienten ist und nichts anweisen kann, also möchte. Was ich gut nachvollziehen kann, weil da die / weil er nicht sich quasi einem Risiko / oder den Patienten in ein Risiko bringen möchte. Aber dafür ist die Struktur einfach nicht geschaffen, sodass der Patient dann quasi an einem Stand stehen bleibt, ohne die Möglichkeit, sich zu bewegen. Ohne eine Klinikeinweisung, die ja oft nicht gewollt ist." (O)<br>• „(…) und da ist die Situation mit den Ärzten (…) sehr gleich - potentiell (…) mit dem Weg in die Hausarztpraxis, ja, gegen ein klinisches Setting und die schwerstbetroffenen Patienten POTENTIELL entscheiden. (…) Weil die Leute ja sich irgendwie auch bewusst gegen / vielleicht auch bewusst gegen diese intensivmedizinischen Patienten entschieden haben (…)." (S)<br>• „(…) ich hatte eine Patientin mit einem gynäkologischen Problem, das ist so gut wie unmöglich, ein gynäkologisches Konsil zu bekommen für jemanden, der eine Trachealkanüle hat, hatte ich das Gefühl. Das war ein ewig langer Prozess." (S)<br>• „Und wir haben die außerklinische Intensivpflege, wo ich das Gefühl habe, man muss ständig hinterherrennen (…), dass man Neurologen überhaupt findet, dass ich einen Zahnarzt überhaupt finde." (S)	Insufficient qualification of physicians regarding the treatment and care of ventilated patients hinders an optimal development of the patients.<br><br>Lacking expertise of a GP regarding the ventilation of patients led to necessary instructions for the optimal care of the patient not being given, thereby making improvements harder to achieve.<br><br>The basic professional training for physicians does not (sufficiently) qualify to work with patients with TC, so that many health care workers do not have the confidence to work with the severely affected patients.

• „Und zwar habe ich das Gefühl, ich habe öfter miterlebt, okay, Patienten haben irgendein Problem. Das entdeckt die Intensiv-WG und macht sich auf die Suche: „Wer kann mir helfen bei dem Problem?“ Prallt beim Arzt ab, prallt beim nächsten Arzt ab und/, also es kümmert sich keiner so richtig. Weil entweder kein Arzt da ist oder vielleicht das Problem auch nicht behoben wird. Und dann ist der nächste Schritt eine Klinikeinweisung. Und die Klinikeinweisung sagt: „Naja, der ist doch bei Euch gerade gut versorgt, wir haben gerade keinen Platz für einen Beatmeten“. Und dann verschlechtert sich die Situation, man läuft wieder zu den Ärzten, versucht irgendjemanden zu finden, der was machen kann, sage ich jetzt mal. Und erst dann, wenn es ein richtig kritischer critical Fall ist, gibt es ein Bett, weil dann die Priorität hoch genug ist.“ (S)<br>• „(…) könnten wir da nicht Vieles irgendwie verbessern, wenn beispielsweise ein festes Ärzteteam bei einer Intensiv-WG sein muss (…). Aber es ist wahrscheinlich genauso wie bei den Therapeuten, es fehlt halt diese flächendeckende Versorgung.“ (S)	It is difficult to obtain a medical consultation for patients with a TC.<br><br>It is difficult to find medical specialists to care for the severely affected patients in the outpatient intensive care residential community. Only when the patient's medical condition becomes so critical that hospitalization is unavoidable can the patient be admitted.<br><br>There is a shortage of physicians to care for the patients in the outpatient intensive care residential community.
Technical aids	
• „Was auch noch manchmal die Umsetzung einer guten Weiterbetreuung verhindert ist, dass zum Teil die Hilfsmittel eben nicht adäquat vorhanden sind.“ (P)<br>• „Aber doch kennen wir das so, dass die Intensiv-WGs nicht die Möglichkeit haben, einen gewissen Pool aufzubauen. Also beispielsweise was wir aus der Klinik kennen, dass ich zwanzig Trachealkanülen auf Vorrat habe in zwanzig verschiedenen Größen, wo ich mal was ausprobieren kann, weil ich sehe, die Trachealkanüle jetzt sitzt nicht. Diese Möglichkeit gibt es gerade nicht in den Intensiv-WGs (…). Doch denke ich, dass es zum Beispiel dazu führen könnte, dass man nicht erst warten muss, bis jetzt der Hilfsmittelversorger liefern kann, und dann ist die falsche geliefert, und, ich glaube, diese Situation haben wir alle schon mal erlebt.“ (S)<br>• „Ansonsten habe ich das Gefühl, dass manchmal Hilfsmittel tatsächlich sehr barrierevoll sind. Es müssen sich Menschen da dahinterklemmen, dass diese Hilfsmittelprozesse überhaupt erfolgreich sind. Das sind AIP-Mitarbeiter, das sind Familienmitglieder, die da dahinter sind und versuchen, mit den Krankenkassen zu verhandeln und zu telefonieren, aber letztendlich gibt es Patienten, die zwei Jahre lang die falsche Bettgröße haben gefühlt. Also, das ist jetzt überspritzt, aber schon einfach lange, lange Hilfsmittelprozesse mit durchaus sinnvollen Dingen. Und im besten Fall kommt er dann, wenn der Patient vielleicht schon zu schlecht geworden ist, weil er VIELLEICHT die Therapie nicht bekommen hat.“ (S)	The problematic situation in the provision of medical or technical aids hinders needs-orientated care, since aids are in some cases not available in the outpatient intensive care residential community, the process up to delivery is sometimes lengthy and complicated and a stock of aids is not funded for the outpatient intensive care residential community.
Networking	
• „Es gibt / ich glaub, in der Organisationsstruktur ist, glaube ich, das größte Problem, was die Umsetzung quasi behindert, dass es dezentral ist, also dass quasi jede Berufsgruppe für sich arbeitet und für sich finanziert wird. (…) Ich sehe viele Einzelkämpfer an den Betten, die dann auch teilweise sehr gute Arbeit sicherlich leisten wollen oder auch können, aber das wird nicht als Team praktiziert (…)“(O)<br>• „Und dass es keine Abspracheverpflichtung zwischen den einzelnen Berufsgruppen gibt und das auch dadurch nicht gefördert wird, genau.“ (O)<br>• „Das sehe aktuell nicht, dass da berufsübergreifend Ziele formuliert werden.“ (O)<br>• „Das Problem, jetzt ist eben, dass es viele verschiedene Dokumente gibt, die auch nicht beim Patienten bleiben, so dass es auch kein interdisziplinär übergreifendes Wissen gibt, wo man mal nachschlagen kann, was zum Beispiel der Arzt warum, wann gemacht hat, oder die Logopädin, warum sie das Entblocken vielleicht verbessert / also erweitert oder auch abgesetzt hat. Und das ist natürlich sehr ungünstig, weil so kann keiner lernen, und so fließen die Informationen nicht.“ (O)	The provision of needs-based care is hindered by the decentralised care structure, in which the individual professional groups receive separate funding and work separately.<br><br>There is no requirement for agreements between the individual professional groups caring for the patients, so that needs-based care cannot be facilitated.<br><br>Goals for the patients are not set across all professions involved in patient care.<br><br>The existence of different documentation systems, which are also not kept at the patient's bedside, prevents access to interdisciplinary information being relevant across all involved professions.

Financing	
• „Ich denke auch, dass wir hier große finanzielle Fehlanreize haben, sowohl für die AKI selber, ich denke, als auch für die anderen Berufsgruppen. Jetzt wenn ich auch an diese neuen Rahmengesetzgebungen denke, die ja darauf abzielen, diese Intervention-/ das Potential der Entwöhnung zu erheben et cetera, et cetera, wo sich wieder neue Strukturen etabliert haben, die sich quasi gegenseitig finanzieren. (…) Und ich denke auch, dass (…) man ein klares Zeichen setzen muss, dass eine / nur die Kanüle als solche nicht die Grundlage einer Pflegebedürftigkeit ist, weil sonst wird nämlich Dekanülierung durch finanzielle Fehlanreize wahrscheinlich nicht stattfinden, auch wenn sie vielleicht möglich wäre, weil natürlich daran die finanzielle Grundlage hängt.“ (O)<br>• „Also manchmal ist es ja so, dass man Leute aus dem Team oder auch vielleicht Angehörige schulen könnte, irgendwelche Dinge auch außerhalb der Therapie zu machen, wenn schon die Therapie es selber nicht leisten kann. Auch das ist abrechnungstechnisch nicht ganz so einfach.“ (S)<br>• „Und ehrlich gesagt sind auch für mich finanzielle Anreize ein Thema (…) es gibt ja auch Eins zu Eins-Versorgung zu Hause. Wenn da eine Therapeutin sagt: „ich mache nur Eins zu Eins-Versorgungen zu Hause“, da müssen die Patienten schon ganz schön nah an der Praxis wohnen, damit sich das finanziell rechnet, wieviel Zeit man im Auto verbringt. Weil mit dieser Fahrtkostenpauschale, die ist halt ein Tropfen auf den heißen Stein. Und gerade in ländlichen Regionen, wo du gut und gerne mal eine halbe, dreiviertel Stunde zum Patienten fahren würdest, kann ich es niemandem verübeln, dass er oder sie sagt: „Nein, mache ich nicht. Also, das fahre ich einfach nicht, oder ich kann es nicht leisten, weil ich einfach in der Zeit genauso gut einen Patienten behandeln hätte können“.“ (S)<br>• „Bürokratie ist ein RIESEN Thema.“ (S)	The structure of the financing of services generates financial disincentives for outpatient intensive care residential community and for other professional groups involved, which leads to a situation in which needs-based care is hindered, for example by patients not being decannulated due to the securing of a financing basis, even though this would be possible.<br><br>To increase the intensity of speech therapy exercises, caregivers and relatives could be supervised by speech therapists, but financing such supervision is difficult.<br><br>The travelling allowance for outpatient therapies is too low, especially in rural areas, so that not all patients in the outpatient intensive care residential community can receive the therapy they need.<br><br>Bureaucracy (volume of documentation/billing) is a major problem in providing needs-based care.

Explanations: O - occupational therapist; P - physician; S - speech- and language therapist; GP - general practitioner; TC – tracheal cannula.

<u>Thematic field:</u> Appropriateness of the clinical pathway for the support of needs-based healthcare (medical and organizational aspects)

<u>Thematic code category:</u> Clinical pathway conceptualization/ positive aspects

Examples of individual statements	Summary statement
• „Also im Prinzip muss ich sagen beschreibt der klinische Pfad das schon bedarfsgerecht. (…) Inhaltlich, wie gesagt, ist da alles drin, was man braucht, um ein Team zusammenzufassen. Vielleicht wären da noch einige Ergänzungen notwendig, damit auch der ärztliche Bereich zum Beispiel komplett abgebildet wird, weil das ist ja jetzt schon eher therapeutisch ausgelegt, aber das könnte man ja alles ergänzen.“ (O)	The CP basically describes needs-based care; further information should be added i.e. for the medical field.

Explanations: O - occupational therapist.

<u>Thematic field:</u> Appropriateness of the clinical pathway for the support of needs-based healthcare (medical and organizational aspects)

<u>Thematic code category:</u> Clinical pathway conceptualization/ negative aspects

Examples of individual statements	Summary statement
• „Also im Prinzip muss ich sagen beschreibt der klinische Pfad das schon bedarfsgerecht. Wir haben bloß momentan ein Problem mit der Umsetzung. (…) Man müsste bloß diese Art der Dokumentation verpflichtend für alle Berufsgruppen und auch am quasi Patientenbett direkt stationieren und dafür alle anderen Dokumentationsgrundlagen entfernen, so dass quasi ein gemeinsames lebendes Dokument erschaffen wird. Und das würde dann halt inhaltlich sowie organisatorisch die Versorgung verbessern, bin ich fest davon überzeugt.“ (O)<br>• „Also, ich sehe ganz oft ganz große Papierordnerakten, wo es verschiedene therapeutische Dokumentationen schon innerhalb von einem Ordner sozusagen gibt (…). Oder wo Lagerungsprotokolle erfasst werden, wo ärztliche Notizen, ein ärztlicher Verlauf einsehbar ist, wo ich schauen kann, wann war das letzte Mal ein Neurologe da, was hat der da reingeschrieben. Mein Gefühl ist, die/der Handschriftlichkeit in allen Ehren, sobald man dann 800 Seiten in einem Leitz-Ordner hat, schaut sich die auch keiner mehr an. Weil man einfach ohne eine Schulung für diesen Ordner auch gar nicht weiß, wo kann ich denn da was nachlesen beziehungsweise wo kann ich /. Also, ich habe auch gar nicht die Zeit, mir die 800 Seiten vorher mal anzugucken. Jetzt bin ich wahrscheinlich damit schon bei Frage 5 und nicht bei Frage 4 (…)“ (S)	There is an insufficient implementation of the CP and the patient folder in the day-to-day routine, which could be addressed by keeping only one single documentation basis at the patient's bedside that is mandatory for all healthcare staff.<br><br>There is too much handwritten documentation.

Explanations: O - occupational therapist; S - speech- and language therapist; CP – Clinical Pathway.

<u>Thematic field:</u> ROFT support for needs-based healthcare <u>Thematic code category:</u> ROFT support/ positive aspects

Examples of individual statements	Summary statement
• „Also, die Arbeitshilfen, die wir an die Hand bekommen haben, waren teilweise nützlich (…). Also ich meine, es ist definitiv gut, die Idee, zum Beispiel des Ordners, also des klinischen Pfades, war eine sehr gute Idee, finde ich.“ (O)<br>• „Andere Dinge, wie zum Beispiel die Möglichkeit der ISSA oder der NIR, denke ich, sind unglaublich wertvolle Werkzeuge, um einem Patienten bei Versorgungsengpässen oder bei akutem Behandlungsbedarf oder einem Weaning-Versuch schnell und gute Hilfe vielleicht dann auch unter klinischen Bedingungen, die man draußen vielleicht durch fehlendes Monitoring nicht etablieren kann, zu gewähren.“ (O)<br>• „Und was ich sehr geschätzt habe, ist dieses zwei bis drei Stunden Zeit für den Patienten zu haben. Also, dahin zu gehen und ganz ausführlich den Patienten untersuchen zu können, mit den verschiedenen Leuten reden zu können, im besten Fall kam eine Therapeutin noch vorbei, mit der ich noch sprechen konnte. Ich konnte mich ganz intensiv mit der Pflege austauschen, und in den zwei bis drei Stunden kann man dann halt auch ein bisschen flexibler reagieren (…) Das, hatte ich das Gefühl, das hat auch irgendwie geholfen, überhaupt in dieser Studie sozusagen / also, eine Flexibilität, die ich jetzt als ambulante Logopädin nie hatte, weil einfach diese Zeit nicht da ist, da vier Stunden für jeden Patienten vor Ort zu sein. Weil das einfach auch nicht bezahlt wird.“ (S)	The work aids and materials provided (such as the patient folder) were useful in part.<br><br>The possibility of inpatient admissions as part of the study for interdisciplinary assessments (ISSA) and neurological interval rehabilitation (NIR), for a planned weaning attempt, in case of acute treatment needs or care gaps, is an important tool for needs-based care.<br><br>A very positive aspect was having two to three hours of time for one patient for detailed assessment, for an exchange with various people and to be able to react flexibly to the day-to-day requirements.

Explanations: O - occupational therapist; S - speech- and language therapist.

<u>Thematic field:</u> ROFT support for needs-based healthcare <u>Thematic code category:</u> ROFT support/ negative aspects

**Examples of individual statements**	**Summary statement**
• „Also, die Arbeitshilfen, die wir an die Hand bekommen haben, waren (…) teilweise aber aufgrund der, ja, der Entfernung, sage ich jetzt mal, und der, sagen wir mal, der Zugriffsgeschwindigkeit manchmal nicht so ganz günstig (…) Aber aufgrund dessen, dass wir nur ein externer Behandler sind und eben NICHT die Möglichkeit haben, Behandlungsanweisungen zu geben, wurde das natürlich einfach zu wenig oder gar nicht genutzt.“ (O)<br>• „Eine andere Vorgehensweise würde ich machen in organisatorischer Hinsicht. Ich glaube, es wäre günstiger gewesen, wenn WIR als AFNTs fünf lokalen WGs (…) als unterstützendes Team quasi also angekoppelt wären, so dass wir quasi relativ schnell agieren können und die Patienten auch regelmäßiger visitieren können (…). Ich glaube, dass ein Vor-Ort-Blick besser ist als der telefonische Austausch, der letztendlich nur zu gegenseitiger Belastung führt.“ (O)<br>• „Wir haben ja die Visiten zu bestimmten Zeiten gemacht und die erste Visite war bereits nach vier Wochen. Das war auch gut so, aber ich würde sogar die Zeit zwischen der Entlassung aus der neurologischen Reha und dieser ersten Visite noch kürzer machen, weil da insbesondere am Anfang der Studie eigentlich am meisten passiert ist. Da sind am meisten (...) irgendwelche schwerwiegenden Ereignisse gewesen, wo die Patienten wieder ins Krankenhaus mussten, irgendwelche Infektionen /. Und da würde ich, glaube ich, die Zusammenarbeit noch intensivieren zwischen so was Ähnlichem wie einem OptiNIV- Team und der Intensiv-WG.“ (P)<br>• „Ja, ich glaube, ein großer Faktor für mich, den ich anders machen würde, ist das Thema Zeit (…). Denn Zeit im Sinne von auch vergüteten Stunden /. Denn ich habe sehr viele super engagierte Menschen kennengelernt, die sich gerne auch MEHR noch ins Projekt eingebracht hätten und sich gerne einmal die Woche Zeit auch genommen hätten für eine interdisziplinäre Teamkonferenz. Aber in dem Moment, wo es einfach für mich überhaupt nicht wirtschaftlich ist und ich diese Stunde aus meiner privaten Zeit opfere, kann ich niemandem verübeln, dass er diese Stunde nicht investiert. Das heißt, ich glaube, ich würde daran / da versuchen, dass man da eine Ausgleichsmöglichkeit macht.“ (S)<br>• „Keine Dopplungen in Dokumenten finde ich ein Thema. Also, wir haben diesen Patientenhefter bei den / am Patientenbett sozusagen etabliert, und trotzdem gibt es noch irgendwo anders einen Leitz-Ordner, wo faktisch manche Dokumente doch ähnlich sind. Das heißt, ich habe viele Therapeut*innen gebeten und gesagt: „Ah, wäre das für Euch okay, wenn Ihr doppelt dokumentiert, einmal bei uns im Ordner und einmal dort, wo Ihr sowieso dokumentiert“. Weil die einfach eine andere Form haben, die sie gerne hätten, die jetzt nicht unserem Patientenhefter entsprochen hätten. Das, also, da verstehe ich auch, dass da Leute dann irgendwann sagen: „Ja, das fand ich ein bisschen blöd“ (…).“ (S)<br>• „Und auch nicht zu viel Papier, weil in dem Moment, wo wir einen Patientenhefter mit 200 Seiten irgendwie da reinlegen, zu dem Leitz-Ordner mit 200 Seiten, wird es ja nur noch mehr Dokumente, durch die man durchsteigen muss.“ (S)<br>• „Und ohne eine Schulung – auch so ein Thema – Schulung für die Dokumente, Schulungen dafür, was passiert überhaupt in diesem Ding, und dafür auch Zeit zu haben. Also, wir haben das versucht, mit einem Anschreiben und so zu lösen, aber letztendlich, wer liest sich auch ein Anschreiben von zwei DIN A4-Seiten durch.“ (S)<br>• „Genau, ansonsten habe ich noch / was ich noch anders machen würde, wäre EIN AFNT für alle Patienten einer WG. Also, ich habe gemerkt, dass es für mich jetzt als Person, die ja komplett neu in diese Intensiv-WG kommt, auch viel Zeit kostet zu verstehen, wie funktioniert die WG, wie laufen die Abläufe, welche Physiotherapeutin macht hier denn was, ja, oder also behandeln alle Physiotherapeutinnen alle Patienten oder wonach gehen diese Dinge. Also, ganz organisatorische Dinge, wo man Nachfragen sozusagen vermeiden kann, indem man einfach selber ein bisschen ein Stück (…) Routine in der WG hat. Und wo ich mir denke, es gibt WGs, mit denen hatte ich eine gute Routine, da habe ich verstanden, wie die Abläufe sind, da kannte ich die Leute, die Leute kannten mich. Und da hätte man nochmal diesen positiven Effekt für mehr Patienten nutzen können, wenn ich nicht nur einen Patienten in jeder WG gehabt hätte, sondern zwei oder drei oder vier. Dann hätte man nicht, ich sage jetzt mal, diese Beziehungsarbeit und die Erfahrungsarbeit, mit was, wie funktioniert diese WG, dieser Pflegedienst, nicht jedes Mal aufs Neue leisten zu müssen, sozusagen. Da hätte mehr Zeit sozusagen dann in die Patienten vielleicht nochmal gehen können.“ (S)	The tools and materials provided (such as the patient file) were used insufficiently due to the difficulty of accessing them for outpatient follow-up care staff and the lack of treatment instructions.<br><br>From an organisational point of view, it is preferable to assign the outpatient specialist aftercare teams to a certain number of local fixed outpatient intensive care residential communities to be able to respond quickly and personally and thus reduce the burden of telephone communication.<br><br>It would be favourable to schedule the first visit by the outpatient specialist aftercare team earlier after discharge from neurological rehabilitation, as the time after discharge is particularly critical.<br><br>t would be helpful to establish a financial compensation option for certain activities, such as attending the interdisciplinary team meetings, to enable the respective team members to become even more involved in the project.<br><br>The duplicate storage of documents with comparable content should be avoided.<br><br>The paper-based patient documentation should be kept short and concise.<br><br>It would have been desirable if training courses on patient documentation had been offered to staff with the corresponding time off.<br><br>It would have been beneficial if only one member of the ROFT had been responsible for all patients in a particular residential community to keep the organisational aspect to a minimum.

Explanations: O - occupational therapist; P - physician; S - speech- and language therapist; ROFT: regional outpatient follow-up team.

<u>Thematic field:</u> Additional aspects for needs-based healthcare <u>Thematic code category:</u> Medical aspects

Examples of individual statements	Summary statement
• „Ich denke, es wäre wichtig, Standards zu haben. Standards, die verpflichtend sind. (…) wir haben zumindest die Beobachtung gehabt (…), dass stationäre Einrichtungen in der Regel einen, ja, vielleicht nicht besseren Komfort bieten, aber zumindest subjektiv eine bessere Sicherheit dem Patienten bieten, weil die dort gegebenen Monitoringanlagen einfach viel besser sind als rudimentäre Babyphone, die da sonst in den Zimmern stehen.“ (O)<br>• „Also, da könnte man auch, wenn man über schwer betroffene Patienten mit Beatmung und so weiterredet, natürlich auch an Sicherheit denken. Und, diese / da müsste man schon drüber nachdenken, ob die Struktur einer Wohngemeinschaft, die ja eigentlich den Sinn hat, dass man ein wohnliches Umfeld schafft, aber da es ja oft nicht gegeben ist, wenn man ehrlich ist, vielleicht nicht doch in einen stationären pflegerischen Kontext zurückgehört.“ (O)<br>• „Und wie gesagt, in der Organisation betone ich, dass ich denke, es wäre gut, wenn man die Betreuung und die Finanzierung dieser Menschen daran orientiert, dass man quasi immer die Salutogenese in den Vordergrund rückt. Dass sie gesund werden dürfen und dass die Finanzierung davon nicht abhängig ist, welchen Zugang sie haben, sondern am Schweregrad ihrer Erkrankung und am Förderbedarf im Alltag orientiert wird.“ (O)	Mandatory standards would be important, i.e. in the safety of ventilated and cannulated patients.<br><br>Consideration should be given to whether outpatient intensive care residential communities, whilst intended to create a homely environment, offer sufficient security for the severely affected ventilated and cannulated patients or whether inpatient nursing care would be more appropriate.<br><br>In general, patient care and the financing of healthcare services should focus on recovery and be orientated towards the severity of the illness and the need for rehabilitation in daily life (and funding not be dependent on the presence of a TC / mechanical ventilation).

Explanations: O - occupational therapist; TC – tracheal cannula.

<u>Thematic field:</u> Additional aspects for needs-based healthcare <u>Thematic code category:</u> Organizational aspects

Examples of individual statements	Summary statement
• „Und ich denke auch, dass die Angehörigen entlastet werden müssten.“ (O)<br>• „(…), dass man Menschen, die zum Beispiel einen Pflegeberuf, Pflegehelfer oder Ähnliches gelernt haben, die Möglichkeit gibt, sich hier weiter zu differenzieren und vielleicht auch weiter auszubilden. Das könnte auch die Möglichkeit sein, wenn man dieses Konzept der weiteren Ausbildung und Supervision und so weiter beherzigt, dass man da mehr Leute dann für diese Berufe auch begeistern kann bzw. auch / ja, letztendlich dann auch in diesem Beruf fördern kann.“ (O)<br>• „Da wäre es dann fast sinnvoll, ein Casemanagement für diese schwer kranken Fälle mit der Komplexität ihrer Erkrankungen und auch der Finanzierung zu haben, die jeden einzelnen Fall fest steuern und da dann auch die Ansprechpartner bündeln.“ (O)<br>• „Ich sehe viele Einzelkämpfer an den Betten, die dann auch teilweise sehr gute Arbeit sicherlich leisten wollen oder auch können, aber das wird nicht als Team praktiziert, sodass auch viele Leistungen ins Leere laufen, auch finanzieller Natur.“ (O)<br>• „Noch auf den Aspekt von – ich sage jetzt mal – diese Notfall- oder Komplikationsmanagement eingehen. (…) Und erst dann, wenn es ein richtig kritischer critical Fall ist, gibt es ein Bett, weil dann die Priorität hoch genug ist. Und da frage ich mich manchmal schon, könnten wir da nicht Vieles irgendwie verbessern, wenn beispielsweise ein festes Ärzteteam bei einer Intensiv-WG sein muss (…) Die sich mit diesen Patienten befassen können, so, oder befassen, so, und das dann tatsächlich auch tun und dann vorbeikommen regelmäßig und sich kümmern und die Patienten kennen.“ (S)	Family members of patients in the shared flats should be given some relief.<br><br>Further training programmes and supervision for professionals in the nursing field could make these professions more attractive and contribute to their professional development.<br><br>Employing a case manager for the severely neurologically affected patients would be useful due to the complexity of the illnesses and (financial) challenges.<br><br>The deployment of a fixed medical team that is responsible for one outpatient intensive care residential community, that knows and regularly visits the patients and provides medical treatment, when necessary, could improve needs-based care.

Explanations: O - occupational therapist; S - speech- and language therapist.
