# Supplementary material for: Post-discharge “continuum of care” clinical pathway (CP) for persons with severe neuro-disabilities – qualitative research to model needs-based community healthcare, capture the real-life care situation, and assess the appropriateness of the CP's concept with input from community- and hospital-based healthcare professionals
Source: Front Neurol. 2026 May 12;17:1677483. doi: 10.3389/fneur.2026.1677483 (PMC13248884; doi:10.3389/fneur.2026.1677483)
Supplement: Supplementary file 5 [file Data_Sheet_5.pdf]

Supplementary Table 5. Summary statements from the three different stakeholder groups home-based specialized intensive care nursing (HSICN), therapists from the community sector (THER-C) and regional outpatient follow-up team (ROFT).

Sequence of presentation (thematic fields):
Description of an appropriate needs-based healthcare
Implementation of needs-based healthcare
Appropriateness of the clinical pathway for the support of needs-based healthcare (medical and organizational aspects)
ROFT support for needs-based healthcare
Additional aspects for needs-based healthcare

<u>Thematic field:</u> Description of an appropriate needs-based healthcare

Stakeholder-group/ Thematic code category	HSICN	THER-C	ROFT
Content-related Aspects			
Aspect “Nurses”	Needs-based nursing care primarily comprises professional nursing staff with basic and advanced training as well as specialist intensive care nurses.	Needs-based care includes more than the minimum of basic nursing care alone.	///
Aspects “Therapists”	A needs-based, multi-professional therapeutic care includes occupational therapy, speech therapy, physiotherapy, psychology, and respiratory therapy, which enable the patient to participate in everyday life and to take part in life.	A needs-based care comprises transdisciplinary therapy and rehabilitation, i.e. speech therapy, occupational therapy, and physiotherapy to provide the patients with the opportunity to improve in the different areas.<br><br>Psychological consultation, dietary counselling, and nutritional medical treatment play an important role in needs-based care.	Needs-based care involves speech- and language therapy, physiotherapy, and occupational therapy treatment.

Aspects "Physicians"	Needs-based medical care primarily comprises GP care, but also care by specialists (neurologists, urologists, ENT specialists, anesthetists, dentists).	///	Needs-based care involves adequate care by physicians.
Aspects "Technical aids"	A needs-based patient care comprises an adequate TC management.	Needs-based care includes monitoring and flexible interventions when required.<br><br>The regular fiberoptic endoscopic evaluation of swallowing (FEES) contributes to needs-based care, as it can accelerate the achievement of goals in the context of dysphagia-management by speech and language therapy treatment.	///
Aspects "Medication"	///	Needs-based care entails that the medication is tailored to the patient's current needs.	///
Organizational aspects			
Aspects "Nurses"	A nursing ratio of 1(nurse):3(patients) is considered necessary to provide good nursing care for the severely affected patients.<br><br>The leadership hierarchy in nursing should be reduced to a single central role to save human resources.<br><br>Local hospitals qualified to care for these critically ill patients are needed. Alternatively, care during hospital stays could be provided in non-specialized facilities by nursing staff in shared living communities (for a fee).	Adequate numbers of qualified care professionals in the residential communities are important for the provision of needs-based care.	Nursing care should be organized in such a way that it is tailored to the specific requirements of these neurologically and pneumological severely ill patients and that it meets the needs of these patients.<br><br>In the area of nursing care, it is important to continuously expand specialist knowledge by participating in continued medical education to be able to provide good care for severely affected patients.

Aspects "Nurses" (continued)			Residential intensive care should be organized in such a way that it includes the professional groups of nursing as well as physicians and therapists on a mandatory basis and that the services for patients are organized centrally and delegated by the medical staff.<br><br>Care for the patients with severe neurological disorders should be provided by non-profit organizations<br><br>The need for care should not be defined by the presence of a tracheal cannula and after decannulation or mechanical ventilation and after weaning alone, i.e. there should be a financing option for continued care of these severely affected patients in the residential homes with intensive nursing care even when mechanical ventilation or tracheal cannula are no, longer needed.
Aspects "Therapists"	Central provision and placement of specialists such as respiratory therapists by companies can support needs-based care and should be carried out by trained specialists as needed.	Setting up a timetable in which the appointments of the outpatient therapists are scheduled would be helpful for a good organization of processes in the residential community.<br><br>Additional therapeutic personnel resources would facilitate needs-based care.	A team of therapists is needed that has the necessary knowledge and training to work with this group of patients with adequate time frames.<br><br>New professions such as respiratory therapists, perhaps with delegation authorization offer the potential to effectively support the difficult care situation in residential intensive nursing care.

Aspects “Therapists” (continued)		Outpatient speech and language therapy treatment should be organized in a way that it can take place with sufficient frequency and on a regular basis for needs-based care.<br><br>The provision of regular reports on medical examinations and an interprofessional exchange of findings and therapy goals support needs-based planning of further speech- and language therapy treatment.	
Aspects “Physicians”	Physicians (especially GPs) should be given the opportunity to complete additional training in outpatient intensive care medicine.<br><br>The timely provision of specialist medical care is also part of a needs-based care.	///	Medical treatment should be provided by internal medicine or neurology consultants with specialist qualifications in intensive care medicine who have experience with this patient group and are able to carry out diagnostic interventions.
Aspects “Technical aids”	Telemedicine would be one method of realizing needs-based care.	///	The supply of medical and technical aids should be organized by the discharging rehabilitation center in a timely manner, supported by the team in the selected residential intensive nursing care home to ensure that all necessary assistive devices are already available on discharge from the rehabilitation center.

Aspects “Networking”	Good co-operation and support between nursing staff and physicians and therapists is regarded as a prerequisite for needs-based care.<br><br>A coordinator and contact person for the team in the residential community, who controls and organizes further medical care, is considered important.<br><br>Leadership and its structures in the residential community should encourage transdisciplinary thinking, the contribution of ideas and a concept-based cooperation within the nursing team.<br><br>The continuation of outpatient care by outpatient specialist aftercare teams would form a valuable addition and can help to prevent hospital admissions.	The most important factor in the realization of needs-based care is that patients are cared for by a multidisciplinary team.<br><br>Good organization, team leadership and management are important factors for successful and efficient teamwork in the residential communities.<br><br>Support from relatives contributes to needs-based care.<br><br>To ensure needs-based care, better coordination, and delegation of decision-making powers for the outpatient care structure is necessary.<br><br>Regular training courses and further measures to develop professional expertise provide positive support for needs-based patient care.<br><br>The teams should be organized in such a way as to allow experienced nurses and therapists to support beginners in the field and that there is always a person on site who has specialized knowledge of TC management.	Minimum standards should be set for professional training and for the work in teams, which should include the topics of collaboration, communication, and goal setting.<br><br>In the initial period after the patient's admission from the rehabilitation clinic to the outpatient intensive care residential community, close support from an outpatient aftercare specialist team, for example provided by the rehabilitation clinic, would be an important factor for needs-based care.

Aspects "Networking" (continued)		Needs-based care means that even severely affected patients - despite the increased time required - are given the opportunity to express their needs and that nurses and therapists in outpatient care are given the opportunity to implement this time commitment.<br><br>Regular team meetings within a fixed team, for example once a week, would be desirable for needs-based care.	
Aspects "Financing"	///	Additional financial resources would facilitate needs-based care.	Cost coverage for the time required for transdisciplinary exchange, intensive preparation for a patient and team training would be a prerequisite for need-based care.

Explanations: GP - general practitioner; TC – tracheal cannula; /// -No statement from the stakeholder group in the relevant code category.

<u>Thematic field</u>: Implementation of needs-based healthcare

Stakeholder-group/ Thematic code category	HSICN	THER-C	ROFT
		Facilitating aspects	
Aspects "Nurses"	A positive, committed work attitude and the ability of nurses to recognize and promote each patient's individual resources contribute to needs-based care.<br><br>Providing compassionate and appreciative care and attention to severely ill patients is part of needs-based care, and the patients' gratitude rewards this work.<br><br>The nursing staffing ratio of 1:3 in the outpatient intensive care residential communities is appropriate based on the existing guidelines.<br><br>The employment of additional care assistants, even without refinancing, supports needs-based care.	Due to the shortage of nursing staff, many geriatric nurses with retraining are being employed in the residential communities.<br><br>A facilitating factor for needs-based care is the good care-to-patient ratio in the residential community.	The implementation of needs-based care is supported by the existing training and further education opportunities for the nursing staff in the outpatient intensive care residential communities.<br><br>Dedicated people (employees in the outpatient intensive care residential communities) are the key to needs-based care.
Aspects "Therapists"	Speech-and language therapy, occupational therapy and physiotherapy of good quality and sufficient intensity facilitates needs-based care.	Guided speech therapy exercises, which were carried out daily by nursing staff, considerably increased the treatment success.	Dedicated people (therapists) are the key to needs-based care.
Aspects "Physicians"	Telemedicine can facilitate specialist medical care when resources are limited.	If outpatient neurologists are unavailable, treatment prescriptions can also be made by other physicians, such as ENT specialists.	///

Aspects "Networking"	Effective collaboration with GPs and specialists promotes needs-based care.<br><br>An effective, trust-based, and patient-centred cooperation with an outpatient specialist aftercare team is a factor that facilitates the needs-based care.<br><br>The realisation of needs-based care is facilitated by committed and supportive cooperation amongst all stakeholders involved in the provision of medical or technical aids.<br><br>Communication training and coaching to promote constructive communication within the team is a prerequisite for needs-based care.<br><br>A team leader in the residential communities who has leadership and management skills and provides optimum support and encouragement to the team also promotes needs-based care.<br><br>Nursing staff can be relieved if certain tasks can be delegated to specialists such as respiratory therapists.	Needs-based care is facilitated by multidisciplinary cooperation of physicians, nursing staff and therapists.<br><br>Team training and other skills development activities lead to improved quality of needs-based care for patients.	The nursing staff in the outpatient intensive care residential communities often become acquainted with the patient during inpatient rehabilitation already and use the opportunity to exchange information with the carers in the clinic, which promotes needs-based care.

Aspects "Financing"	Organising the patient care in outpatient intensive care residential communities offers various organisational, financial and personnel advantages compared to care in the home environment.	The provision of outpatient intensive care shared living facilities supports needs-based care because patients can live together and several residents can be cared for during therapeutic home visits in a travel- and cost-efficient manner.	There is an overall good financial situation for the care of the severely neurologically affected patients in Germany with a satisfying standard in terms of accommodation and equipment.
		Barriers	
Aspects "Nurses"	In the absence of a central contact person, the nursing staff in the outpatient intensive care residential communities must deal with the challenging task of finding and retaining medical specialists to care for the patients.<br><br>The recruitment of suitable nursing staff poses a challenge.	There is a shortage of nursing staff in the outpatient residential communities.<br><br>A lack of specialised knowledge on the part of nursing staff is a barrier to needs-based care for the patients.	Insufficient qualification of nurses regarding the treatment and care of ventilated patients hinders an optimal development of the patients.<br><br>There is a shortage of nursing staff - a situation where more job positions are available than can be filled (due to income and working conditions), being relevant to society.
Aspects "Therapists"	The need for outpatient speech- and language therapists cannot be fully met.	A lack of specialised knowledge on therapists is a barrier to needs-based care for the patients.<br><br>There is a shortage of speech- and language therapists specializing in in outpatient dysphagia and TC management, meaning many patients cannot receive the treatment they need. Reasons i.e. fear of a lack of expertise and the therapeutic risk.	Insufficient qualification of therapists regarding the treatment and care of ventilated patients hinders an optimal development of the patients.<br><br>There is a shortage of therapists- a situation where more job positions are available than can be filled (due to income and working conditions), being relevant to society.

Aspects "Therapists" (continued)		The frequency of speech therapy treatment is not sufficient.	The basic professional training for therapists does not (sufficiently) qualify to work with patients with tracheal cannula, so that many health care workers do not have the confidence to work with the severely affected patients.<br>Time required to read the extensive medical reports in preparation for the therapeutic treatment of a new patient with a complex medical background is not remunerated separately.
Aspects "Physicians"	For certain medical issues of neurologically severely affected patients, general practitioners often lack the specialist knowledge and options to provide optimal treatment.<br><br>Outpatient medical specialists who accept the severely affected patients as part of their medical care are difficult to find, especially in rural areas.	A missing contact with the physicians and a lack of prescriptions for swallowing diagnostics to monitor progress and plan further treatment make it difficult to provide needs-based speech- and language therapy.<br><br>Home visits from medical specialists are difficult to obtain for patients in outpatient intensive care.	Insufficient qualification of physicians regarding the treatment and care of ventilated patients hinders an optimal development of the patients.<br><br>Lacking expertise of a GP regarding the ventilation of patients led to necessary instructions for the optimal care of the patient not being given, thereby making improvements harder to achieve.<br><br>The basic professional training for physicians does not (sufficiently) qualify to work with patients with tracheal cannula, so that many health care workers do not have the confidence to work with the severely affected patients.<br><br>It is difficult to obtain a medical consultation for patients with a tracheal cannula.

Aspects "Physicians" (continued)			It is difficult to find medical specialists to care for the severely affected patients in the outpatient intensive care residential community. Only when the patient's medical condition becomes so critical that hospitalization is unavoidable can the patient be admitted.<br><br>There is a shortage of physicians to care for the patients in the outpatient intensive care residential community.
Aspects "Technical aids"	Providing patients with medical or technical aids is challenging due to the health insurance companies' restrictive authorisation procedures.	As using the communication board is time-consuming and strenuous, the patients' need to communicate can often not be satisfied in stressful everyday situations.	The problematic situation in the provision of medical or technical aids hinders needs-orientated care, since aids are in some cases not available in the outpatient intensive care residential community, the process up to delivery is sometimes lengthy and complicated and a stock of aids is not funded for the outpatient intensive care residential community.
Aspects "Medication"	///	Prescriptions for medication and their adaptations are not as optimal in the outpatient residential communities as they are in hospitals.	
Aspects "Networking"	Necessary acute hospital admissions to local hospitals can be associated with secondary risks, as these hospitals are not specialised for the treatment of neurologically severely affected people.	The necessary coordination and delegation of authority for the outpatient professions is not sufficient.	The provision of needs-based care is hindered by the decentralized care structure, in which the individual professional groups receive separate funding and work separately.

Aspects "Networking" (continued)	Weaning attempts without an transdisciplinary supporting team, as the outpatient specialist aftercare teams in the OptiNIV study, are very unlikely to be feasible in the familiar environment of the outpatient intensive care residential communities.<br><br>The need of subacute patients in outpatient intensive care residential communities for rehabilitation stays is not fully covered.	Poor coordination, delegation, and teamwork on the part of the physician providing medical care in two residential communities makes it difficult to deliver needs-based care to the patients.<br><br>Overlaps between therapy sessions and care activities despite fixed and regular therapy appointments make it difficult to provide needs-based (speech- und language therapy) care.<br><br>There are no time resources for activities that go beyond direct (speech- and language) therapy work with the patient, e.g. for exchanging information with other therapists or reading medical reports.<br><br>It is a lengthy process from organising an appointment with the physician in the residential community to being sent the medical results.<br><br>At some hospitals, attempts are made to avoid admitting the severely neurologically affected patients from outpatient intensive care.	There is no requirement for agreements between the individual professional groups caring for the patients, so that needs-based care cannot be facilitated.<br><br>Goals for the patients are not set across all professions involved in patient care.<br><br>The existence of different documentation systems, which are also not kept at the patient's bedside, prevents access to transdisciplinary information being relevant across all involved professions.
Aspects "Financing"	Cost negotiations with payers regarding reimbursement for care ratios and services are difficult and complicate patient care. To provide needs-based care, facilities	Due to the lack of cost coverage by health insurance companies, necessary nursing consumables cannot be reordered in sufficient quantities.	The structure of the financing of services generates financial disincentives for outpatient intensive care residential community and for other professional groups involved, which leads to a

Aspects "Financing" (continued)	must provide more than they are reimbursed for.<br><br>Financial reasons may result in a patient not being decannulated, even though this would be possible from a medical point of view.<br><br>Once a patient is admitted to the hospital for weaning from a TC or ventilation, the outpatient intensive care residential communities no longer receive funding. However, they must keep the care bed free for that patient and cannot accompany them to the hospital.<br><br>The potential of the patients is often not seen during hospitalisation, and the care of these severely affected patients is typically highly cost-intensive.<br><br>Home visits by medical specialists and GP in the residential communities are not adequately refinanced.<br><br>Not all nursing services can employ additional respiratory therapists and specialist staff at their own expense unless they are externally funded by health insurance companies.	One barrier to needs-based care is that the costs of certain treatments (such as osteopathy and alternative medical treatments) are not covered by statutory health insurances.<br><br>Due to limited medical care options in smaller community hospitals hospital treatments may be required in larger. urban hospitals, which places a greater burden on the patient due to longer transport distances and higher costs.	situation in which needs-based care is hindered, for example by patients not being decannulated due to the securing of a financing basis, even though this would be possible.<br><br>To increase the intensity of speech therapy exercises, caregivers and relatives could be supervised by speech therapists, but financing such supervision is difficult.<br><br>The travelling allowance for outpatient therapies is too low, especially in rural areas, so that not all patients in the outpatient intensive care residential community can receive the therapy they need.<br><br>Bureaucracy (volume of documentation/billing) is a major problem in providing needs-based care.

Explanations: GP - general practitioner; TC – tracheal cannula; /// -No statement from the stakeholder group in the relevant code category.

<u>Thematic field</u>: Appropriateness of the clinical pathway for the support of needs-based healthcare (medical and organizational aspects)

Stakeholder-group/ Thematic code category	HSICN	THER-C	ROFT
Implementation reflection, clinical pathway / Positive aspects	A positive aspect is the patient-centred collaboration between the different professional groups involved in patient care described in the clinical pathway.<br><br>A common, informative documentation system for all persons involved in patient care, to which everyone has access, is considered useful.	A documentation system should present the status and objectives of the patient in a clear, compact, and standardised way for all professional groups involved in patient care in a folder, thus making it possible to obtain the information briefly in a time-saving manner.<br><br>Providing contact information of other professionals involved in care as part of the documentation would be useful.<br><br>Team conferences could be held online via video-calling.	The CP basically describes needs-based care; further information should be added i.e. for the medical field.
Implementation reflection, clinical pathway / Negative aspects	Duplicate or unnecessary documentation should be avoided to save resources.	Communication between therapeutic professions can be improved through written documentation. However, a uniform documentation system would need to be implemented to avoid duplication of documentation.	There is an insufficient implementation of the CP and the patient folder in the day-to-day routine, which could be addressed by keeping only one single documentation basis at the patient's bedside that is mandatory for all healthcare staff.<br><br>There is too much handwritten documentation.

Explanations: CP – Clinical pathway.

<u>Thematic field</u>: ROFT support for needs-based healthcare

Stakeholder-group/ Thematic code category	HSICN	THER-C	ROFT
Positive aspects	It was helpful to be supported by accessible, committed, and reliable contact persons, to receive useful advice for patient care and to have the opportunity to enhance knowledge during the team visits.<br><br>A useful aspect is, that certain diagnostic tests and check-ups could be conducted in the residential community, thus avoiding hospitalisation.	Medical care has been improved in the residential community through the work of the outpatient aftercare study team.<br><br>The documentation for the study was positive, despite the need for double documentation during this period.<br><br>The regular phone calls and follow-up enquiries from the outpatient aftercare study team constitute a positive aspect.<br><br>The collaboration with the outpatient aftercare study team was very pleasant, interesting, and helpful.<br><br>The study's regular swallowing examinations provided more certainty and opportunities for the planning and execution of the speech therapy treatment, which could accelerate decannulation.<br><br>The opportunity for transdisciplinary contact and exchange with neurologists and speech therapists during the study was helpful.	The work aids and materials provided (such as the patient folder) were useful in part.<br><br>The possibility of inpatient admissions as part of the study for transdisciplinary assessments (ISSA) and neurological interval rehabilitation (NIR), for a planned weaning attempt, in case of acute treatment needs or care gaps, is an important tool for needs-based care.<br><br>A very positive aspect was having two to three hours of time for one patient for detailed assessment, for an exchange with various people and to be able to react flexibly to the day-to-day requirements.

Negative aspects	The additional activities of an external outpatient specialized aftercare team are not efficient if the team in the shared living community already covers all aspects of care.<br><br>Due to a lack of information about planned study visits, interested team members of the outpatient intensive care communities were sometimes unable to participate in the visits and facilitate transdisciplinary collaboration on-site.<br><br>Emails are more suitable for conveying the necessary patient information in the busy day-to-day care setting than the required daily phone calls.<br><br>Outpatient care is not optimal if treatment recommendations lead to a deterioration in the patient's condition and hospital admissions.<br><br>Due to a lack of communication interfaces, the nursing staff of the outpatient intensive care community was unable to exert the desired influence on the care of their patients during hospital stays.	According to information from the nursing staff, the study and the associated daily calls from the outpatient aftercare study team were sometimes perceived by the nursing staff as very time-consuming and annoying.<br><br>There was a negative experience with a FEES of a patient, planned and carried out by the outpatient aftercare study team, where the arrangements and organisation with the outpatient intensive care team were not optimal.	The tools and materials provided (such as the patient file) were used insufficiently due to the difficulty of accessing them for outpatient follow-up care staff and the lack of treatment instructions.<br><br>From an organisational point of view, it is preferable to assign the outpatient specialist aftercare teams to a certain number of local fixed outpatient intensive care residential communities to be able to respond quickly and personally and thus reduce the burden of telephone communication.<br><br>It would be favourable to schedule the first visit by the outpatient specialist aftercare team earlier after discharge from neurological rehabilitation, as the time after discharge is particularly critical.<br><br>It would be helpful to establish a financial compensation option for certain activities, such as attending the transdisciplinary team meetings, to enable the respective team members to become even more involved in the project.<br><br>The duplicate storage of documents with comparable content should be avoided.

Negative aspects (continued)			The paper-based patient documentation should be kept short and concise.<br><br>It would have been desirable if training courses on patient documentation had been offered to staff with the corresponding time off.<br><br>It would have been beneficial if only one member of the ROFT had been responsible for all patients in a particular residential community to keep the organisational aspect to a minimum.

<u>Thematic field:</u> Additional aspects for needs-based healthcare

Stakeholder group/ Thematic code category	HSICN	THER-C	ROFT
Medical aspects	Every potential for improvement must be utilized and valued to improve the patient's quality of life.<br><br>Prerequisites for needs-based care include creating a positive environment for the patient, a good relationship with the nurse, and communicating all necessary information, such as planned interventions.<br><br>It would be desirable for the treating hospital to be interested in the patient's further development in the outpatient intensive care unit after discharge, as the initial period after discharge is particularly crucial for the patient's further development.<br><br>It is advisable to measure treatment success qualitatively in different areas rather than focussing on goal achievement 'decannulated' or 'weaned'. The achieved improvements by the study patients could thus be shown.	Patients can also benefit from the work of other therapeutic professionals such as osteopaths, alternative practitioners, or massage therapists.	Mandatory standards would be important, i.e. in the safety of ventilated and cannulated patients.<br><br>Consideration should be given to whether outpatient intensive care residential communities, whilst intended to create a homely environment, offer sufficient security for the severely affected ventilated and cannulated patients or whether inpatient nursing care would be more appropriate.<br><br>In general, patient care and the financing of healthcare services should focus on recovery and be orientated towards the severity of the illness and the need for rehabilitation in daily life (and funding not be dependent on the presence of a TC / mechanical ventilation).

Organizational aspects	Cooperation with relatives is one important aspect in the care of patients in the outpatient intensive care residential communities<br><br>Patients have expressed the wish to be cared for in the outpatient intensive care residential communities. and with no hospitalisations.<br><br>Participation of study patients in the interviews would have been desirable.<br><br>Further study projects would promote needs-based care in that the group of the severely affected patients in the outpatient intensive care residential communities would receive more attention.<br><br>An intensive interval rehabilitation relatively early after initial neurorehabilitative treatment would be desirable to optimise the progress from the patients in the outpatient intensive care residential communities.<br><br>During hospital stays, the nursing staff of the outpatient intensive care residential community wish to exert further influence on the treatment of patients.	Cost coverage by health insurance companies and the associated possibility of supplying medical and care aids is severely limited and therefore in need of improvement.<br><br>Within the context of needs-based care, patients could receive more treatments from therapists with diverse professional backgrounds.	Family members of patients in the shared flats should be given some relief.<br><br>Further training programmes and supervision for professionals in the nursing field could make these professions more attractive and contribute to their professional development.<br><br>Employing a case manager for the severely neurologically affected patients would be useful due to the complexity of the illnesses and (financial) challenges.<br><br>The deployment of a fixed medical team that is responsible for one outpatient intensive care residential community, that knows and regularly visits the patients and provides medical treatment, when necessary, could improve needs-based care.

Organizational aspects (continued)	There should be an organisational body that mediates between the institutions involved (hospital, outpatient intensive care residential community, perhaps GP, and medical specialists).<br><br>The continuation of the OptiNIV study would benefit the needs-based care of patients in outpatient intensive care communities and could lead to cost reductions.		

Explanations: TC - tracheal cannula.
